# Targeting ST8SIA6-AS1 counteracts KRAS^G12C^ inhibitor resistance through abolishing the reciprocal activation of PLK1/c-Myc signaling

**DOI:** 10.1186/s40164-023-00466-3

**Published:** 2023-12-16

**Authors:** Yafang Wang, Mingyue Yao, Cheng Li, Kexin Yang, Xiaolong Qin, Lansong Xu, Shangxuan Shi, Chengcheng Yu, Xiangjun Meng, Chengying Xie

**Affiliations:** 1 Shanghai Institute for Advanced Immunochemical Studies, ShanghaiTech University, 393 Middle Huaxia Road, Shanghai, 201210 People’s Republic of China; 2 Division of Life Sciences and Medicine, The First Affiliated Hospital of USTC (Anhui Provincial Hospital), University of Science and Technology of China, Hefei, Anhui China; 3 Drug Discovery and Development Center, Shanghai Institute of Materia Medica, Chinese Academy of Sciences, 555 Zuchongzhi Road, Shanghai, 201203 People’s Republic of China; 4 School of Life Science and Technology, ShanghaiTech University, Shanghai, 201210 China; 5 Lingang Laboratory, 319 Yueyang Road, Shanghai, 200031 China; 6 Gastroenterology, Shanghai Ninth People’s Hospital, Shanghai Jiao Tong University School of Medicine, Shanghai, 200001 China; 7 China Center for Digestive Diseases Research and Clinical Translation of Shanghai Jiao Tong University, Shanghai, 200001 China; 8 China Shanghai Key Laboratory of Gut Microecology and Associated Major Diseases Research, Shanghai, 200001 China

**Keywords:** KRAS^G12C^, c-Myc, PLK1, LncRNA, ST8SIA6-AS1, Drug resistance

## Abstract

**Background:**

KRAS^G12C^ inhibitors (KRAS^G12C^i) AMG510 and MRTX849 have shown promising efficacy in clinical trials and been approved for the treatment of KRAS^G12C^-mutant cancers. However, the emergence of therapy-related drug resistance limits their long-term potential. This study aimed to identify the critical mediators and develop overcoming strategies.

**Methods:**

By using RNA sequencing, RT-qPCR and immunoblotting, we identified and validated the upregulation of c-Myc activity and the amplification of the long noncoding RNA ST8SIA6-AS1 in KRAS^G12C^i-resistant cells. The regulatory axis ST8SIA6-AS1/Polo-like kinase 1 (PLK1)/c-Myc was investigated by bioinformatics, RNA fluorescence in situ hybridization, RNA immunoprecipitation, RNA pull-down and chromatin immunoprecipitation. Gain/loss-of-function assays, cell viability assay, xenograft models, and IHC staining were conducted to evaluate the anti-cancer effects of co-inhibition of ST8SIA6-AS1/PLK1 pathway and KRAS both in vitro and in vivo.

**Results:**

KRAS^G12C^i sustainably decreased c-Myc levels in responsive cell lines but not in cell lines with intrinsic or acquired resistance to KRAS^G12C^i. PLK1 activation contributed to this ERK-independent c-Myc stability, which in turn directly induced PLK1 transcription, forming a positive feedback loop and conferring resistance to KRAS^G12C^i. ST8SIA6-AS1 was found significantly upregulated in resistant cells and facilitated the proliferation of KRAS^G12C^-mutant cancers. ST8SIA6-AS1 bound to Aurora kinase A (Aurora A)/PLK1 and promoted Aurora A-mediated PLK1 phosphorylation. Concurrent targeting of KRAS and ST8SIA6-AS1/PLK1 signaling suppressed both ERK-dependent and -independent c-Myc expression, synergistically led to cell death and tumor regression and overcame KRAS^G12C^i resistance.

**Conclusions:**

Our study deciphers that the axis of ST8SIA6-AS1/PLK1/c-Myc confers both intrinsic and acquired resistance to KRAS^G12C^i and represents a promising therapeutic target for combination strategies with KRAS^G12C^i in the treatment of KRAS^G12C^-mutant cancers.

**Supplementary Information:**

The online version contains supplementary material available at 10.1186/s40164-023-00466-3.

## Introduction

KRAS mutations occur in nearly 30% of human cancers, among which about 14.5% are G12C mutations. In recent years, breakthroughs in KRAS-based structure discoveries and mechanism studies lay the conceptual foundation for the clinical development of covalent and selective KRAS^G12C^ inhibitors (KRAS^G12C^i), which have shown great efficacy in early clinical trials [1–4]. However, the therapeutic benefit of the approved KRAS^G12C^i such as Sotorasib (AMG510) and Adagrasib (MRTX849), and other drugs under development is limited by the intrinsic resistance or the rapid emergence of acquired resistance in nearly all the treated KRAS^G12C^-mutant tumors including non‑small cell lung cancer (NSCLC), pancreatic adenocarcinoma (PAAD) and colon adenocarcinoma (COAD). By far, the reported mechanisms include KRAS^G12C^ secondary activation mutation [5], acquired bypass or accessory pathway activation such as RTK/SHP2/MAPK and PI3K/AKT pathway [6], proto-oncogene activation or tumor suppressor gene inactivation [7], etc., indicating that KRAS-mutant tumors have a high degree of genomic heterogeneity. Accordingly, the search for common sensitive indicators and overcoming strategies for both intrinsic and acquired resistance to KRAS^G12C^i is of great significance. A genomic and histologic analyses comparing pretreatment samples with those from patients who developed acquired resistance to KRAS^G12C^i showed that focal MYC amplification exists in both pre- and post-resistant biopsies [8]. And overexpression of c-Myc in PAAD confers both primary and acquired chemoresistance to the combined trametinib plus hydroxychloroquine therapy [9]. However, the exact role and regulatory mechanisms of c-Myc elevation in KRAS^G12C^i- resistant tumors have not been fully elucidated yet.

Long noncoding RNAs (lncRNAs, longer than 200 nucleotides) are transcribed widely from the human genome, and have been identified to be a critical layer of biological regulation via cooperating with DNA, RNA or protein partners [10–12]. Although a variety of lncRNAs exhibit abnormal expression in human cancers and are associated with tumor progression [13] and poor patient outcomes [14], only a few have been functionally characterized. The molecular mechanisms underlying their pleiotropic biology remain largely unknown. Notably, a previous study reported that KRAS overexpression induces numerous responsive lncRNAs including KIMAT1, which is found essential for KRAS-driven cancer cell survival and reciprocally potentiates KRAS signaling [15]. Whereas, the mechanistic relevance of lncRNA signaling involved in KRAS^G12C^i resistance is poorly understood by now. In view of the recent promising outcomes with RNA-based therapeutics [16, 17], it is worthwhile to identify the resistance-related lncRNAs and their downstream network for therapeutic purpose to halt the adaptation to KRAS^G12C^i.

Here, we illustrated that c-Myc alteration monitors the response to KRAS^G12C^i treatments and further elucidated the mechanistic role of the hyper-activated lncRNA ST8SIA6-AS1/Aurora A/PLK1 signaling in conferring c-Myc stability and KRAS^G12C^i resistance. Our data depicts the existence of a positive feedback regulatory loop that enhances the oncogenic axis of ST8SIA6-AS1/PLK1/c-Myc and provides complementary targets for therapeutic interventions. Combined targeting of this axis and KRAS effectively abrogates drug resistance, causing cell death and tumor regression. These findings help to provide important rationales for suppressing ST8SIA6-AS1/PLK1/c-Myc pathway in combination with KRAS^G12C^i, when treating resistant KRAS^G12C^-mutant tumors.

## Methods

### Cell culture

Human cancer cell lines (NCI-H358, NCI-H1373, NCI-H23, SW1573, MIA PaCa-2, SW1463) were initially purchased from the American Type Culture Collection (ATCC, Manassas, VA) and cultured in a humidified atmosphere (37 °C, 5% CO_2_ or 100% air). All these cells were maintained following the providers’ instructions. The culture conditions were described in Additional file 1: Table S1. Murine pro-B cell line Ba/F3 transfected with plasmids expressing KRAS^G12C^ or KRAS^G12C^ secondary mutants were grown in RPMI 1640 with 10% fetal bovine serum; otherwise supplemented with 10% WEHI-3-conditioned medium as a source of interleukin-3 in the case of parental cells. 293T cells were grown in DMEM medium (Thermo Fisher Scientific). All cell lines were routinely confirmed for Mycoplasma negativity and authenticated using short tandem repeat (STR) method based on the ATCC profiles.

### Cell viability assay

Cells were seeded at an appropriate density of 3000–5000 per well in 96-well plates and maintained overnight before exposure to gradient doses of the drugs. After treatments, cell proliferation was analyzed by sulforhodamine B (SRB) assay [18] for adherent cells or methyl thiazolyl tetrazolium (MTT) assay [19] for suspended cells. IC_50_ values were calculated using GraphPad Prism software (La Jolla, CA) and presented in histograms with mean ± SD. For assessing synergistic effect, cells were treated with KRAS^G12C^i, PLK1i alone or in combination at gradient concentrations for 3 days and cell viability was determined by SRB assay. Drug synergy was calculated as the combination index (CI) using CalcuSyn Version 2.0 software [20]: CI < 1, synergy; CI = 1, additive effect; CI > 1, antagonism.

### Western blot assay

To prepare cell lysates, cells after the treatment were washed with cold phosphate buffer saline (PBS) and lysed in RIPA buffer supplemented with protease and phosphatase inhibitors cocktail (100×). For preparation of tissue lysates, tumor tissues from mice were homogenized in RIPA buffer and protein quantification was performed with the BCA Protein Assay Kit (Thermo Fisher Scientific). Equal amounts of proteins were separated on SDS-PAGE gels and transferred onto nitrocellulose membranes. The bands were probed with primary antibodies which were listed in the Additional file 1: Table S2 at 4 °C overnight, then incubated with species-specific HRP-conjugated secondary antibodies from Invitrogen. Chemiluminescence images were captured by Tanon4600 using Pearce ECL Substrate (Thermo Fisher Scientific).

### Small interfering RNA (siRNA) and plasmids transfection

Cells were transfected with 5 nM siRNA using Lipofectamine RNAiMAX transfection reagent (Thermo Fisher Scientific). SiRNAs targeting c-Myc or PLK1 were designed and synthesized by Tsingke. SiRNAs targeting ST8SIA6-AS1 or a scrambled sequence were designed and synthesized by RiboBio. Detailed sequences for siRNAs were listed in the Additional file 1: Table S3. FUW-tetO-hMYC (Cat#: 20723), pCDH-puro-cMyc (Cat#: 46970) and pcDNA3-PLK1(829) (Cat#: 39845) plasmids were obtained from Addgene (Watertown, USA). pcDNA3.1-ST8SIA6-AS1 was constructed by Beyotime Biotechnology (Shanghai, China). For transient-expression vectors, pcDNA3-PLK1(829) or pcDNA3.1-ST8SIA6-AS1 was transfected into cells using Lipofectamine 2000 (Invitrogen, Waltham, MA, USA) according to the manufacturer’s instructions. For stable cell line constructions, lentiviruses were produced in 293T cells transfected with pCDH-puro-cMyc or FUW-tetO-hMYC using TurboFectin transfection reagent (OriGene). Two days after transfection, virus in the supernatant was collected, centrifuged for 5 min, and filtered through 0.45 μm filtration unit (Millipore). The virus was added into cell cultures and positive clones were selected using appropriate antibiotics: zeocin (Beyotime, T1450, 600 µg/mL) or puromycin (Thermo Fisher Scientific, A1113803, 1 µg/mL).

### RNA extraction and quantitative real-time PCR (RT-qPCR)

After treatment, cells were lysed by Trizol and whole-cell RNA was extracted. cDNA was synthesized using PrimeScript™ RT Master Mix (Takara). Nuclear and cytoplasmic RNA was purified according to the manufacturer’s recommendations of the PARIS RNA Purification Kit (Thermo Fisher Scientific). RT-qPCR was performed using the TB Green® Premix Ex Taq™ II (Takara). Specific primers were synthesized by Tsingke, and their sequences were available in the Additional file 1: Table S4. GAPDH was used as an internal control.

### RNA-seq processing

Total RNAs were extracted from MIA PaCa-2 parental and resistant cells using TRIzol® Reagent (Invitrogen). RNA quality was determined by 2100 Bioanalyser (Agilent) and quantified using the ND-2000 (NanoDrop Technologies). RNA-seq transcriptome library was acquired by TruSeqTM RNA sample preparation Kit from Illumina (San Diego, CA) using 1 µg of total RNA and sequenced by Majorbio company (Shanghai, China). FASTQ files were aligned to human reference genome (version: GRCH38.p13; source: http://asia.ensembl.org/Homo_sapiens/Info/Index). Clean reads were separately aligned to reference genome with orientation mode using HISAT2 (http://ccb.jhu.edu/software/hisat2/index.shtml) software. Differential expression analysis was performed using the DESeq2 39 (q value ≤ 0.05, DEGs with |log2FC| > 1 were considered to be significantly differentially expressed genes). The Gene set enrichment analysis (GSEA) was then performed belonging the output to the collection of ‘‘Hallmark’’ gene sets part of the Molecular Signatures Database (MSigDB v7.0, http://software.broadinstitute.org/gsea/downloads.jsp). The threshold for statistical significance chosen in the GSEA was False Discovery Rate (FDR) < 0.25.

### Annexin V/propidium iodide (PI) apoptosis assay

Cell apoptosis after treatment was detected quantitatively using the Annexin V-FITC/PI double-staining apoptosis detection kit (Vazyme Biotech, Nanjing, China). Cells were collected and dyed according to the instruction, and then assayed on the CytoFLEX (Beckman, Pasadena, California, USA). The proportion of apoptotic cells was analyzed by the FlowJo software.

### Cell cycle assay

Briefly, cells were collected and fixed by cold 70% ethanol overnight, followed by RNase A digestion and PI staining at room temperature for 30 min, and then detected by the CytoFLEX. Data from 10,000 cells were acquired in general, and the percentages of cells in each cell cycle phase were analyzed with the BD Cell Quest Pro modify software.

### Colony formation assay

Cells were seeded at the density of 5000–10,000 cells per well in 6-well plates with indicated treatments for 6–8 days until the colonies were visible. Resistance or sensitivity to KRAS^G12C^i was assessed by treating cell lines with KRAS^G12C^i alone or in combination with PLK1i or MYCi975. After washed with PBS, colonies were then fixed in 4% paraformaldehyde at 4 °C for 15 min, and stained with 0.1% crystal violet in methanol for 15 min. Then colonies were washed and dried at room temperature. Colony formation ability was quantitatively measured by dissolving the crystal violet in 33.3% (v/v) acetic acid and detecting the absorbance at 600 nm wavelength on the microplate reader TECAN (Carolina, US).

### Chromatin immunoprecipitation (CHIP) assays

The CHIP assay was carried out with the SimpleCHIP® Plus Enzymatic Chromatin IP Kit (Cell Signaling) using specific antibody against c-Myc or normal rabbit IgG antibody. The assay was performed according to the manufacturer’s recommendations. Briefly, 5 × 10^6^ cells were cross-linked for 15 min by 1% formaldehyde and stopped by 1/20 volume of 2.5 M glycine. Then the cells were scraped from the plate and lysed. The chromatin is fragmented by partial digestion with micrococcal nuclease and subsequently sonicated to obtain chromatin fragments of 1 to 5 nucleosomes in size. Antibody incubations were carried out overnight at 4 °C. Reversal of protein-DNA cross-links was carried out at 65 °C for 2 h. The enrichment of particular DNA sequences was analyzed by quantitative PCR. The sequences of the primers were listed in Additional file 1: Table S4.

### Protein–RNA interaction predictions

The CatRAPID algorithm (http://s.tartaglialab.com/page/catrapid_group) [21] and RNA–protein interaction prediction database (RPISeq, http://pridb.gdcb.iastate.edu/RPISeq/) [22] were applied to estimate the binding propensity of PLK1-ST8SIA6-AS1 interaction. The PROMO (https://alggen.lsi.upc.es/cgibin/promo_v3/promo/promoinit.cgi?dirDB=TF_8.3) [23] was used to identify the binding site of c-Myc on the *PLK1* promoter.

### RNA pull-down assay

The RNA pull-down experiment was performed under RNase-free conditions all through as previously reported [24]. ST8SIA6-AS1 or antisense ST8SIA6-AS1 was transcribed and labeled with Biotin RNA Labeling Mix (RiboBio Biotech, China). Approximately 1 × 10^7^ MIA PaCa-2-R or SW1573 cells were lysed on ice for 10 min with 500 µL polysome extraction buffer [100 mM KCl, 5 mM MgCl_2_, 5% NP40, 1 mM DTT, 1 mM EDTA and 20 mM Tris-HCl (pH7.4)], containing protease and phosphatases inhibitor cocktail (Invitrogen). The cell lysates were collected and incubated with 1–2 µg streptavidin beads coated with biotin-labelled RNA probes at 4 °C overnight to form RNA–protein complexes. After washing, the precipitated RNA–protein mixture was eluted in SDS loading buffer for western blot analyses.

### RNA immunoprecipitation

RNA immunoprecipitation (RIP) was performed using the EZ-Magna RIP kit (Millipore) [25]. In brief, MIA PaCa-2-R cells were lysed with RIP lysis buffer supplemented with RNase inhibitor and protease inhibitor, subjected to one freeze-thaw cycle and centrifuged. Cell extracts were co-immunoprecipitated with PLK1 or Aurora A antibody overnight at 4 °C followed by incubation with protein A/G beads at 4 °C for 4 h. After extensive washing, the bead–protein–RNA mixture was digested with proteinase K at 55 °C for 1 h. The immunoprecipitated RNA remained was retrieved and purified for RT-qPCR analysis. Normal rabbit IgG was used as a negative control in the immunoprecipitation process.

### Cell immunofluorescence and fluorescence in situ hybridization (FISH)

MIA PaCa-2-R were seeded onto coverslips. After treatments, the cells were fixed with 4% paraformaldehyde for 15 min and permeabilized with 0.1% Triton-X100 for 8 min. For cell immunofluorescence, after blockade in 1% BSA for 30 min, the cells were incubated with the p-Histone H3 antibody overnight at 4℃. For immuno-FISH assays [26], permeabilized cells were washed with 2× saline sodium citrate (SSC) and incubated with ~ 0.5 µg of biotin-labeled ST8SIA6-AS1 antisense probe (TTTATCATTCTCTGGCACA, GenePharma, China) in a moist chamber at 37 °C overnight. The following day, cells were washed thoroughly in 2× SSC followed by 1× PBS and then blocked in 1% BSA for 1 h. Then cells were incubated with a mouse anti-biotin antibody along with a rabbit anti-PLK1 antibody overnight at 4 °C. After incubation, cells were washed with 1× PBS and then incubated with a goat anti-mouse Alexa 594 antibody and a goat anti-rabbit Alexa 488 antibody for 1 h. Cells were then washed, stained with 4′,6-diamidino-2-phenylindole (DAPI) (Beyotime) for nuclei detection and finally imaged under a Leica LMS710 laser-scanning confocal microscope (Leica Microsystems, Germany).

### Protein stability detection

The cycloheximide (CHX) chase assay was carried out to detect the protein half-life as previously reported with modifications [27]. Briefly, 20 µg/mL CHX (MedChemExpress) was added for 30, 60, 90 and 120 min in MIA PaCa-2-R cells after transfected with siPLK1 or pcDNA3.1-ST8SIA6-AS1 for 48 h, respectively. To detect the protein degradation pathway, 10 µM MG132 (MedChemExpress) was used to block proteasome-dependent degradation. Western blot was further performed to examine the protein level. Negative controls, involving the use of corresponding negative siRNA or vector, were used in each experimental set.

### Electrophoretic mobility super-shift assay (EMSA)

Biotin-labeled double-stranded oligonucleotide probes corresponding to the DNA sequences containing the E-box site (5′-AGGCTATCCCACGTGTTCGG-3′) on the human *PLK1* promoter, were individually incubated with or without nuclear protein extracts, in the presence or absence of a specific unlabeled cold probe, for 20 min at 4 °C. For the super-shift assay, the nuclear protein extracts were first incubated with c-Myc antibody for 30 min at 4 °C, followed by incubation with the biotin-labeled probe for another 20 min. The mixes were electrophoresed on an 8% non-denaturing polyacrylamide gel and subsequently transferred onto a nylon membrane. The membrane was incubated with streptavidin-conjugated horseradish peroxidase (HRP) for 30 min, washed carefully, and then scanned on Tanon4600 using Pearce ECL Substrate (Thermo Fisher Scientific).

### Luciferase reporter assay

The transcription factor c-Myc-responsive element E-box on the promoter of human *PLK1* gene and an additional mutant promoter fragment were amplified by PCR and subcloned into an enhancer reporter vector, pGL6 (D2102, Beyotime). Mutant promoter in which the binding sequence for c-Myc (proximal CACGTG at − 75 to − 81 nt) was mutated to AACGTG. All recombinant vectors were verified by DNA sequencing.

1 µg of pGL6-basic, PLK1 wild-type (WT) or mutant promoter constructs were co-transfected with 250 ng of Renilla luciferase-expressing control vector pRL-TK (E2241, Promega) into 293T or MIA PaCa-2 cells for 24 h. Cells were lysed with 1× passive lysis buffer (E1941, Promega) and subjected to TACAN microplate luminometer for luciferase activity determination using a Dual-Luciferase reporter assay system (E1910, Promega). The Firefly luciferase activity corresponding to a specific promoter construct was normalized to the Renilla luciferase activity of the same sample. Results were shown as fold changes compared with the mean Firefly/Renilla ratio of the cells transfected with a basic vector.

### Immunohistochemistry (IHC) assay

Tumor tissues were dissected and fixed with 4% paraformaldehyde. IHC staining was performed according to following procedures. Paraffin embedding, sectioning and staining were performed by Zuocheng Company (Shanghai, China). After antigen retrieval and blocking, the slides were incubated with primary antibodies at 4 °C overnight, followed by HRP-conjugated secondary antibodies at room temperature for 1 h, and peroxidase activity was detected with diaminobenzidine (DAB, DAKO). The antibodies used were listed as following: c-Myc (Abcam, Cambridge Biomedical Campus, Cambridge, UK, ab32072); p-ERK (CST, Beverly, MA, USA, Cat #4370); cleaved caspase-3 (CST, Cat #9661). The images were taken at 200× magnification using Tissue FAXS Plus Basic.

### Mouse models and treatment

Female nude mice (Balb/c nude, 5–6 weeks old) were purchased from Charles River Laboratories (Beijing, China). All in vivo experiments were carried out according to the institutional ethical guidelines on animal care and were approved by the Institute of Animal Care and Use Committee at the Shanghai Institute of Materia Medica. Single-cell suspensions were implanted subcutaneously into the right flank of the mice.

The mice were randomly assigned to the control or treatment groups (five mice per group) and started dosing when average tumor volume reached approximately 100 mm^3^. MRTX849 (90% 0.5% CMCNa + 5% PEG400 + 5% DMSO), Volasertib (75% 0.5% CMCNa + 5% DMSO + 10% PEG400 + 10% Tween80) and ST8SIA6-AS1 siRNA (#2 sequence, saline solution) were prepared as the doses indicated. Mice were treated for 3–4 weeks as follows: (i) 0.1% DMSO in normal saline by oral administration once daily; (ii) 20 mg/kg Volasertib by oral administration once per week; (iii) MRTX849 30 mg/kg by oral administration once daily for SW1573 xenografts; 10 mg/kg for MIA PaCa-2 and MIA PaCa-2-R xenografts; (iv) Volasertib plus KRAS^G12C^i co-treatment group. For ST8SIA6-AS1 siRNA-treated groups, the siRNAs (5 nM) were administered by intratumor injection every 3 days. Tumor volumes were measured every 3 days by microcaliper and calculated as follows: ½ × length × width^2^. Body weight was measured twice or three times per week. Mice were euthanized and tumor tissues were collected 2 h after the last dosing and prepared for immunoblotting or immunohistochemistry staining.

### Statistical analyses

All statistical methods used are described in the Figure legends or corresponding methods. In brief, all grouped data are analyzed by the GraphPad Prism software and presented as mean ± standard deviation (SD) or standard error of the mean (SEM). IC_50_ values were determined using a nonlinear regression analysis fit of the normalized dose–response curves. Statistical significance was calculated by two-tailed unpaired student’s t-test for comparisons between two independent groups, oneway ANOVA followed by Tukey’s multiple comparison test for univariate comparisons, and the Pearson coefficient for the linear correlation between two different parameters. Overall survival of lung cancer or pancreatic cancer patients in relation to PLK1 or ST8SIA6-AS1 levels was evaluated by Kaplan–Meier survival curve and 2-sided log-rank test based on the The Cancer Genome Atlas (TCGA) transcriptomics profiles. P < 0.05 was considered statistically significant. All experiments were repeated a minimum of three times.

## Results

### ERK-independent c-Myc signal predicts for the resistance to KRAS^G12C^i

**Fig. 1:**
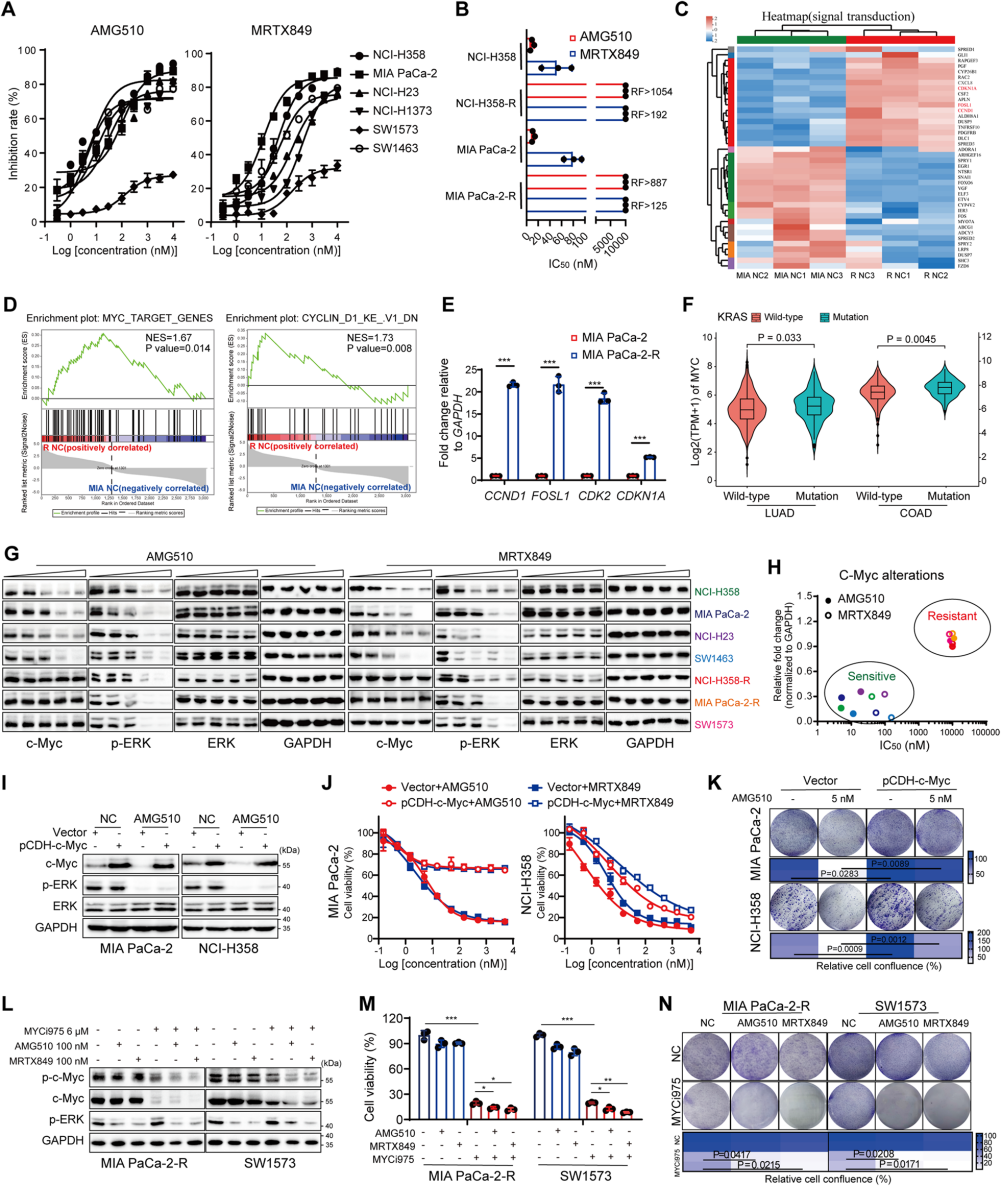
ERK-independent c-Myc signal predicts for the response to KRAS^G12C^i and contributes to KRAS^G12C^i resistance. **A** The cytotoxicity of AMG510 and MRTX849 in six human KRAS^G12C^-mutant cell lines were tested by sulforhodamine B (SRB) assay (n = 3). **B** IC_50_s of AMG510 and MRTX849 matching parental and resistant cells. **C** The hierarchical clustering and heatmap showed the expression profile of the differentially expressed genes (DEGs) in signal transduction pathway from MIA PaCa-2 parental and resistant cells. **D** GSEA showed that gene sets related to MYC targets (left) and CYCLIN D1 signaling (right) were enriched in the KRAS^G12C^i-resistant cells. **E** The mRNA levels of the MYC target genes were tested by RT-qPCR. **F** Expression of c-Myc in LUAD (n = 511) and COAD (n = 402) patients with wild-type or mutant KRAS from TCGA database. The indicated P-values were analyzed by Wilcoxon test. **G** Western blot analysis of KRAS^G12C^-mutant cell lines after dose-dependent (0, 1, 10, 100, 1000 nM) treatment of AMG510 or MRTX849. **H** Association between c-Myc alteration upon KRAS^G12C^i treatment for 24 h versus KRAS^G12C^i sensitivity. Shown were the quantitative results of immunoblotting data in **G**. **I** Western blot analysis of protein lysates from c-Myc-overexpressed MIA PaCa-2 and NCI-H358 cells cultured with or without 100 nM AMG510 for 24 h. **J** Relative cell viability of c-Myc- or vector- expressing MIA PaCa-2 and NCI-H358 cells treated with increasing concentrations of KRAS^G12C^i for 72 h. **K** Colony formation of cells transfected with vector or pCDH-c-Myc plasmid and treated with or without 5 nM AMG510. **L** Western blot analysis of protein lysates from MIA PaCa-2-R and SW1573 cells cultured with KRAS^G12C^i in combination with MYCi975 for 24 h. **M** Relative cell viability of MIA PaCa-2-R and SW1573 cells treated with 1 µM KRAS^G12C^i in combination with 6 µM MYCi975 for 5 days was analyzed by SRB assay (n = 3). **N** Colony formation of cells cultured with 1 µM KRAS^G12C^i in combination with 2 µM MYCi975. Data represent the average and standard deviation (SD). Statistical significance was assessed using two-tailed unpaired Student’s t test. *P < 0.05, **P < 0.01, ***P < 0.001. See also Additional file 2: Figs. S1, S2

To interrogate the molecular features that distinguish KRAS^G12C^i sensitive and resistant cells, we first determined the sensitivity of a panel of six KRAS^G12C^-mutant NSCLC, PAAD and CRC cell lines to KRAS^G12C^i, AMG510 and MRTX849. Most cell lines exhibited sensitivity with half-maximal inhibitory concentrations (IC_50_s) less than 100 nM, whereas SW1573 was not obviously impacted by either treatment, indicating intrinsic resistance (Fig. 1A). The acquired AMG510-resistant MIA PaCa-2-R and NCI-H358-R cells were generated by exposing to sequential increases of AMG510 and displayed resistance to MRTX849 as well (Fig. 1B). Then, MIA PaCa-2 parental and resistant cell lines were subjected to RNA sequencing (RNA-seq) analyses to reveal the signaling dynamics associated with resistance. Heatmap in Additional file 2: Fig. S1A demonstrated that there were significant alterations in the transcriptional program of MIA PaCa-2-R over parental cells. KEGG analysis revealed that signal transduction was the most significantly enriched pathway (Additional file 2: Fig. S1B), among which MYC target genes like *CCND1*, *FOSL1*, and *CDKN1A* were greatly upregulated in MIA PaCa-2-R cells than those in parental cells (Fig. 1C). Further gene set enrichment analyses (GSEA) demonstrated positive correlations of MYC targets and CYCLIND1-dependent signatures with KRAS^G12C^i resistance (Fig. 1D). Real time quantitative PCR (RT-qPCR) analysis validated the increased mRNA levels of MYC target genes in MIA PaCa-2-R cells (Fig. 1E). By investigating the transcriptomic data from The Cancer Genome Atlas (TCGA) dataset, we validated a statistically significant increase of c-Myc expression in KRAS mutant lung adenocarcinoma (LUAD, n = 356) and COAD (n = 173), compared to KRAS wild-type LUAD (n = 155) and COAD (n = 229) respectively, indicating that c-Myc amplification was positively correlated with KRAS mutation in cancer (Fig. 1F). Depletion of c-Myc by specific siRNAs strongly reduced the clonogenic growth of both KRAS^G12C^i sensitive and insensitive cell lines (Additional file 2: Fig. S1C), with reduction of its targets CDK2 and Cyclin D1 expression, whereas elevation of p21 and cleaved caspase-3 levels (Additional file 2: Fig. S1D). All these demonstrated a crucial role of c-Myc for KRAS-mutant cancer growth and drug resistance.

As a critical downstream effect of RAS/MAPK signaling inhibition [28], c-Myc level was obviously reduced under KRAS^G12C^i treatment in KRAS^G12C^i-sensitive cell lines, whereas only modestly affected or remained unchanged in cell lines with intrinsic (SW1573) or acquired resistance (NCI-H358-R and MIA PaCa-2-R) (Fig. 1G, H and Additional file 2: Fig. S1E). In addition, it was noticeable that the degree of ERK suppression after dose- or time-dependent KRAS^G12C^i treatment was not associated with sensitivity to KRAS^G12C^i (Fig. 1G and Additional file 2: Fig. S1E). We also constructed resistant cultures of Ba/F3 cell line transformed with KRAS^G12C^. The Ba/F3 KRAS^G12C^-R cell line showed marked tolerance to KRAS^G12C^i (Additional file 2: Fig. S1F) and a similar phenomenon under the treatment of KRAS^G12C^i was observed: c-Myc levels were only downregulated in Ba/F3 KRAS^G12C^ parental cells, when ERK was inhibited indiscriminately (Additional file 2: Fig. S1G). Accordingly, we found that KRAS^G12C^i treatment following c-Myc knockdown restored the sensitivity in resistant cells (Additional file 2: Fig. S2A). And the combination led to more intense induction of G0/G1 cell cycle arrest (Additional file 2: Fig. S2B) and cell apoptosis (Additional file 2: Fig. S2C, D). On the contrary, forced expression of c-Myc by a pCDH vector, which was not affected by AMG510 (Fig. 1I), permitted MIA PaCa-2 and NCI-H358 cells treated with KRAS^G12C^i to proliferate and anchorage-dependently grow, whereas a null vector failed to rescue such cells (Fig. 1J, K). We next investigated the anti-proliferative effects of c-Myc inhibitors on KRAS^G12C^-mutant cells using MYCi975, a small molecule that directly targets c-Myc [29]. In all the tested KRAS^G12C^i sensitive and resistant cell lines, c-Myc levels were reduced by MYCi975 treatment, and much lower when in combination with AMG510 or MRTX849 (Fig. 1L and Additional file 2: Fig. S2E). Cell proliferation and colony formation were inhibited by MYCi975, and more deeply suppressed under co-treatment with KRAS^G12C^i (Fig. 1M, N and Additional file 2: Fig. S2F, G). All these observations extended prior studies [30] and proposed that the activation of ERK-independent c-Myc signal may be a cause of resistance to KRAS^G12C^i and provide a vulnerability for combinational therapy.

### The reciprocal activation of PLK1/c-Myc pathway confers resistance to KRAS^G12C^i

**Fig. 2:**
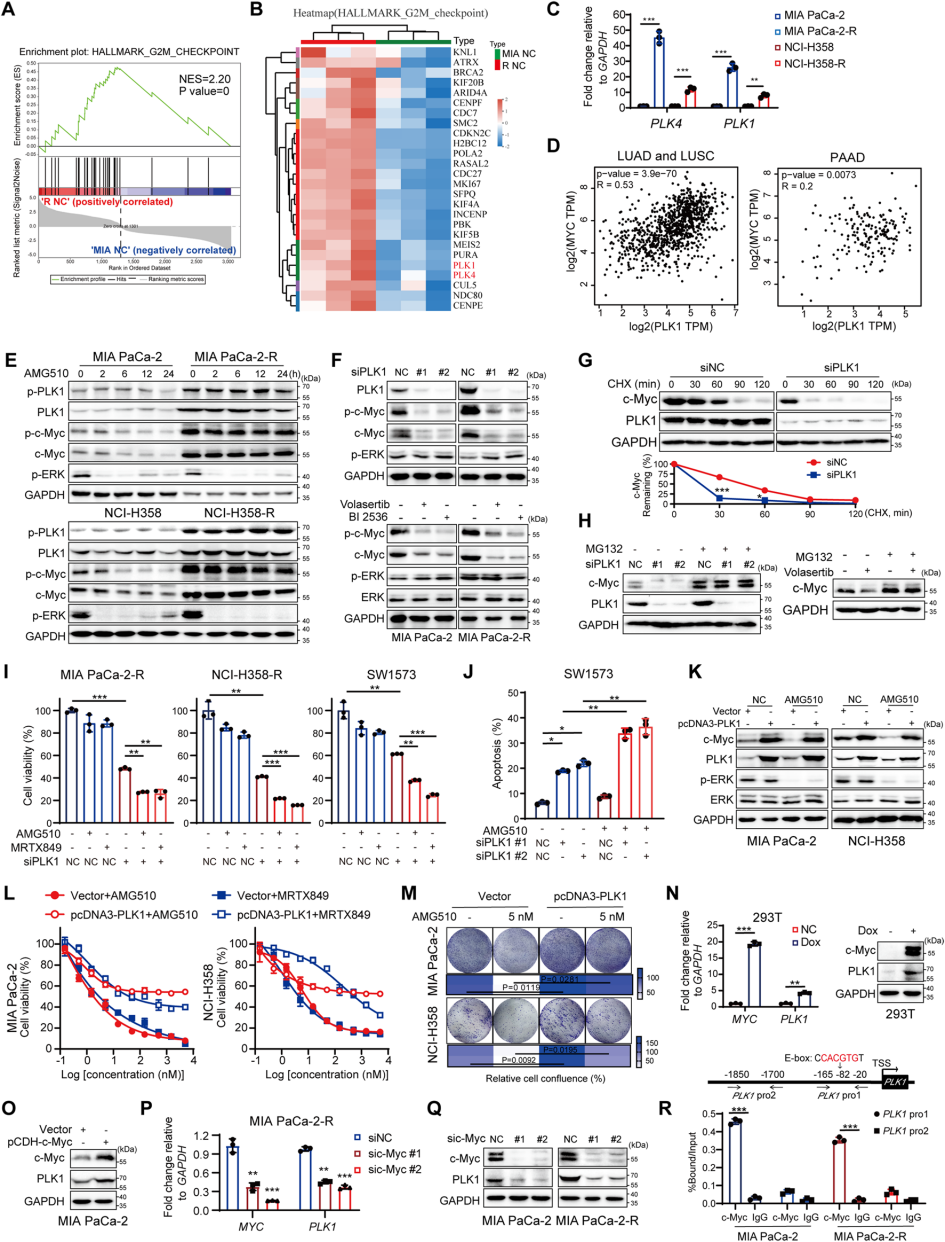
The reciprocal activation of PLK1/c-Myc pathway associates with KRAS^G12C^i resistance. **A** GSEA displayed the gene set related to Hallmark G2/M checkpoint. **B** The hierarchical clustering and heatmap of the gene expressions related to G2/M checkpoint. **C** The mRNA levels of *PLK4* and *PLK1* in MIA PaCa-2 or NCI-H358 parental and resistant cells were tested by RT-qPCR. **D** PLK1 expression is positively correlated with MYC expression in lung and pancreatic cancer patients from GEPIA2 database. **E** Western blot analysis of MIA PaCa-2 or NCI-H358 parental and resistant cells after time-dependent treatments of 100 nM AMG510. **F** Western blot analysis of cells treated with siPLK1s or 20 nM PLK1i for 48 h. **G** MIA PaCa-2-R cells transfected with siNC or siPLK1 were treated with 20 µg/mL CHX for different times and harvested for western blot. The protein abundance of c-Myc was quantified using Image J. **H** C-Myc protein levels were restored by MG132 (10 µM) treatment (6 h) in PLK1-knockdown or Volasertib-treated MIA PaCa-2-R cells. **I** Cell viability of untreated or KRAS^G12C^i-treated (1µM, 72 h) MIA PaCa-2-R, NCI-H358-R and SW1573 cells with PLK1 knockdown (n = 3). **J** Cell apoptosis of PLK1-knockdown SW1573 cells exposed to 100 nM AMG510 for another 24 h. **K** PLK1-overexpressed MIA PaCa-2 and NCI-H358 cells were cultured with and without 100 nM AMG510 for 24 h and subjected to western blot analysis. **L** AMG510 or MRTX849 dose-response curve for PLK1- or vector- expressing cells (n = 3). **M** Clonogenic growth of MIA PaCa-2 and NCI-H358 cells transfected with mock or pcDNA3-PLK1 plasmid and treated with or without 5 nM AMG510. **N** mRNA and protein levels of MYC and PLK1 were assessed under basal conditions or Dox induction (0.5 µM/mL) for 12 h in 293T cells expressing FUW-tetO-hMYC. **O** Western blot analysis of MIA PaCa-2 cells transfected with vector or pCDH-c-Myc plasmid. mRNA (**P**) and protein (**Q**) levels of MYC and PLK1 were assessed in control or c-Myc-knockdown cells. **R** Schematic diagram showed that a c-Myc E-box binding site (CACGTG) existed about 80 bp upstream of the *PLK1* transcription start site (TSS, GeneBank accession number: NC_000016.10) (upper panel). ChIP immunoprecipitation of c-Myc recruitment to the proximal (pro1) and distal (pro2) regions of *PLK1* promoter was analyzed and normalized to the input in KRAS-mutant cells (lower panel). Data represent mean ± SD. Statistical significance was assessed using two-tailed unpaired Student’s t test. *P < 0.05, **P < 0.01, ***P < 0.001. See also Additional file 2: Fig. S3

To define the mechanism underlying the biological effects of KRAS^G12C^i resistance, GSEA of the RNA-seq data revealed that the HALLMARK related genes of G2/M checkpoint were one of the most enriched and up-regulated pathways in MIA PaCa-2-R cells (Fig. 2A). The differentially expressed genes (DEGs) of the G2/M checkpoint components were shown in the heatmap (Fig. 2B) and the concerned genes including Polo-like kinase 4 (PLK4) and PLK1 were highlighted. PLK1, a conserved serine/threonine protein kinase, is activated and phosphorylated at Thr210 by Aurora kinase A (Aurora A) during the G2/M transition [31]. On account of its critical participation in mitotic and non-mitotic processes [31, 32], PLK1 is established as a favorable therapeutic target for cancer therapy. In line with the RNA-seq data, RT-qPCR analysis confirmed the markedly increased mRNA levels of *PLK4* and *PLK1* in resistant cells (Fig. 2C). Elevated PLK1 expression was negatively correlated with overall survival (OS) of PAAD and lung cancer patients analyzed from TCGA database (Additional file 2: Fig. S3A). PLK1 depletion remarkably impaired the cell viability relative to siRNA controls (Additional file 2: Fig. S3B). As an important mitotic checkpoint kinase, PLK1 suppression caused severe mitotic catastrophe, leading to G2/M phase-arrest (Additional file 2: Fig. S3C) and apoptotic cell death (Additional file 2: Fig. S3D). Together, these results suggested that PLK1 is important for KRAS^G12C^-mutant cancer progression.

It’s known that PLK1 directly interacts with c-Myc and induces its S62 phosphorylation [33]. Thus, we intended to clarify whether PLK1 activation was responsible for KRAS^G12C^i resistance through c-Myc stabilization. Data from GEPIA2 showed that PLK1 expression was positively correlated with MYC in lung and pancreatic cancer (Fig. 2D). Through western-blot analyses on several KRAS^G12C^-mutant cell lines, we validated that high basal and phosphorylation levels of PLK1, as well as c-Myc were maintained in KRAS^G12C^i-resistant cells, while downregulated in KRAS^G12C^i-treated sensitive controls (Fig. 2E and Additional file 2: Fig. S3E). Knockdown of PLK1 by siRNAs or pharmacological inhibition by small-molecule inhibitors (PLK1i): Volasertib or BI 2536 in KRAS^G12C^-mutant cells led to dramatic decrease of c-Myc and S62 phosphorylation, which was independent of ERK phosphorylation (Fig. 2F and Additional file 2: Fig. S3F). We found that PLK1 suppression in MIA PaCa-2-R cells attenuated c-Myc half-life dramatically (Fig. 2G) through proteasomal-dependent degradation, which could be rescued by proteasome inhibitor MG132 (Fig. 2H), but not MYC mRNA reduction (Additional file 2: Fig. S3G). PLK1 knockdown re-sensitized KRAS^G12C^-resistant cells to AMG510 or MRTX849 treatment (Fig. 2I and Additional file 2: Fig. S3H). KRAS^G12C^i treatment led to more cell death in PLK1-depleted SW1573 and NCI-H358 cells (Fig. 2J and Additional file 2: Fig. S3I, J), enhancing levels of G2/M cycle arrest markers: Cyclin B1 and phosphorylated histone H3 (serine 10), and an apoptotic marker cleaved caspase-3 (Additional file 2: Fig. S3K). Conversely, exogenously expressed PLK1 sustained c-Myc protein in KRAS^G12C^i-sensitive cells MIA PaCa-2 or NCI-H358 under KRAS^G12C^i treatment (Fig. 2K), which made these cells tolerant to KRAS^G12C^i as seen in cell viability assay (Fig. 2L) and colony formation assay (Fig. 2M and Additional file 2: Fig. S3L).

Then we intended to determine how PLK1 expression was upregulated in KRAS^G12C^i-resistant cells. A study revealed that c-Myc directly activates PLK1 transcription in double-hit lymphoma [34]. We therefore queried whether the observed c-Myc elevation reciprocally induced PLK1 expression. As shown in Fig. 2N, in FUW-tetO-hMYC-transfected 293T cells, the notable induction of c-Myc by doxycycline (Dox) led to marked increases of PLK1 mRNA and protein levels. PLK1 level was also raised by forced-expression of c-Myc in MIA PaCa-2 cells (Fig. 2O). Conversely, c-Myc depletion significantly reduced PLK1 mRNA and protein levels (Fig. 2P, Q). Moreover, as a canonical c-Myc E-box-binding site was identified at the proximal region upstream of the *PLK1* transcription start site (TSS) (Fig. 2R), we performed ChIP assays using both MIA PaCa-2 sensitive and resistant cells and uncovered a significant enrichment of c-Myc binding to the E-box motif when compared to a distal promoter region and IgG isotope control (Fig. 2R). In support of this, EMSA using a biotin-labeled probe containing E-box element, together with nuclear protein extracts of MIA PaCa-2-R cells, detected a strong DNA-protein complex band. The addition of anti-c-Myc antibody resulted in a super-shift of the band, indicating binding of c-Myc to the E-box site on *PLK1* promoter. In contrast, the intensity of the DNA-protein complex band was overtly curtailed using a specific unlabeled cold probe (Additional file 2: Fig. S3M). Moreover, in FUW-tetO-hMYC-transfected 293T cells treated with Dox, the luciferase reporter construct containing a mutant E-box site did not significantly enhance basal promoter activity as the wild-type (WT) E-box construct did. And the luciferase activity dropped dramatically in c-Myc-depleted MIA PaCa-2 cells compared to the control (Additional file 2: Fig. S3N). All these suggested that PLK1 promoted the stability of c-Myc protein, and in turn, c-Myc directly enhanced PLK1 transcription, establishing a positive feedback circuit in KRAS-mutant cancer cells. The acquisition of this highly activated PLK1/c-Myc/PLK1 axis serves as a critical pathway that drives resistance to KRAS^G12C^i.

### PLK1i attenuate KRAS-mutant cell growth and enhance KRAS^G12C^i cytotoxicity

**Fig. 3:**
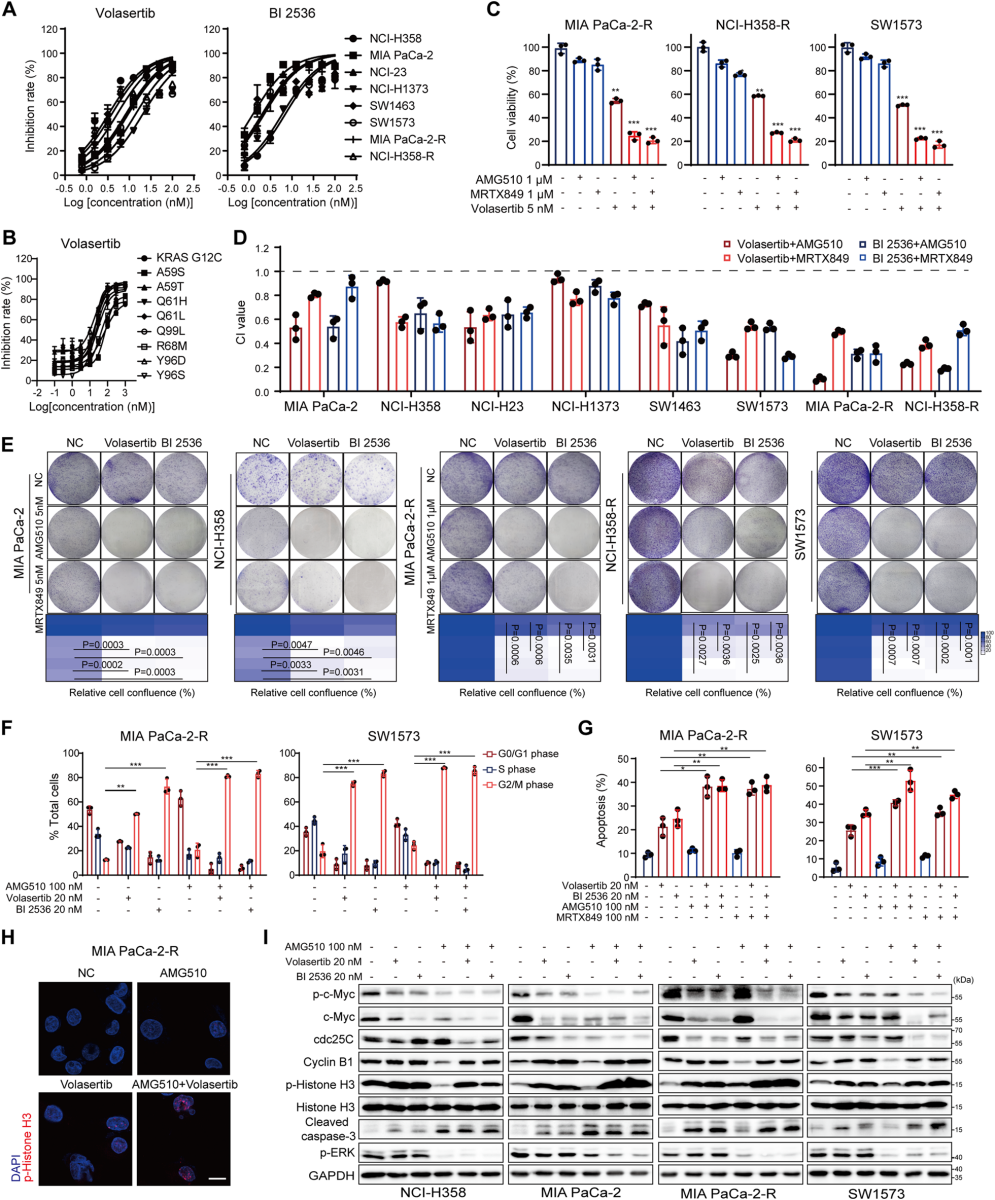
PLK1i synergizes with KRAS^G12C^i via ERK-dependent and -independent downregulation of c-Myc. **A** Representative Volasertib and BI 2536 dose-response curves and IC_50_ values of eight cell lines. **B** Volasertib inhibited the viability of Ba/F3 cells expressing KRAS^G12C^, or KRAS^G12C^ second-site mutants. **C** Relative cell viability of MIA PaCa-2-R, NCI-H358-R and SW1573 cells treated with Volasertib (5 nM), KRAS^G12C^i (1 µM), or their combination for 72 h. **D** CI values of PLK1i in combination with KRAS^G12C^i were tested by cell viability assay and calculated by CalcuSyn software. **E** Clonogenic growth of cells treated with Volasertib (2 nM) or BI 2536 (1 nM) in combination with KRAS^G12C^i. Quantified results were shown. Cell cycle (**F**) and apoptosis analysis (**G**) of cells treated with PLK1i in combination with KRAS^G12C^i for 24 h. **H** Effects of Volasertib (20 nM), AMG510 (100 nM), or their combination (24 h) on histone H3 (Ser10) phosphorylation were shown by immunofluorescence in MIA PaCa-2-R cells. Scale bar, 5 μm. **I** Immunoblotting of cells treated with PLK1i in combination with AMG510 for 24 h. Data represent mean ± SD. Statistical significance was assessed using two-tailed unpaired Student’s t test. *P < 0.05, **P < 0.01, ***P < 0.001. See also Additional file 2: Fig. S4

Given the potent role of PLK1 in sustaining tumor growth [32, 35] and mediating KRAS^G12C^i resistance, PLK1i would serve as both a pharmacological means to interrogate the dependence on PLK1 kinase activity and a promising point for a potential drug combination campaign. As shown in Fig. 3A, PLK1i Volasertib or BI 2536 strongly inhibited the proliferation of both KRAS^G12C^i sensitive and resistant cells with IC_50_ values ranging from 2 to 10 nM. Moreover, Volasertib significantly suppressed cell proliferation of Ba/F3 cells expressing KRAS^G12C^ or KRAS^G12C^ second-site mutants, which have been reported to confer acquired resistance to KRAS^G12C^i [5, 36] (Fig. 3B). These observations proved a previous notion that RAS-mutant cells are hypersensitive to the inhibition of PLK1 function [37]. We then evaluated the synergistic ability of PLK1i and KRAS^G12C^i in overcoming resistance. In the resistant cells, co-treatment of KRAS^G12C^i and Volasertib resulted in more severe decline in cell viability compared to monotherapy (Fig. 3C). And synergistic effects were observed and indicated by the combination index (CI) lower than 1 in various KRAS^G12C^-mutant cell lines (Fig. 3D). The combinational therapies impaired cell long-term colony formation (Fig. 3E) and caused cell death through G2/M cell cycle arrest and apoptosis in both KRAS^G12C^i resistant (Fig. 3F, G) and sensitive cells (Additional file 2: Fig. S4A–D). Immunofluorescence followed by confocal microscopy observation of cells stained with DAPI and histone H3 (serine 10) phosphorylation (p-Histone H3), a mitotic marker [38], confirmed the increase of abnormal cell mitosis after Volasertib and the combined treatment, which indicated that the mitotic accumulation occurred in metaphase (Fig. 3H).

**Fig. 4:**
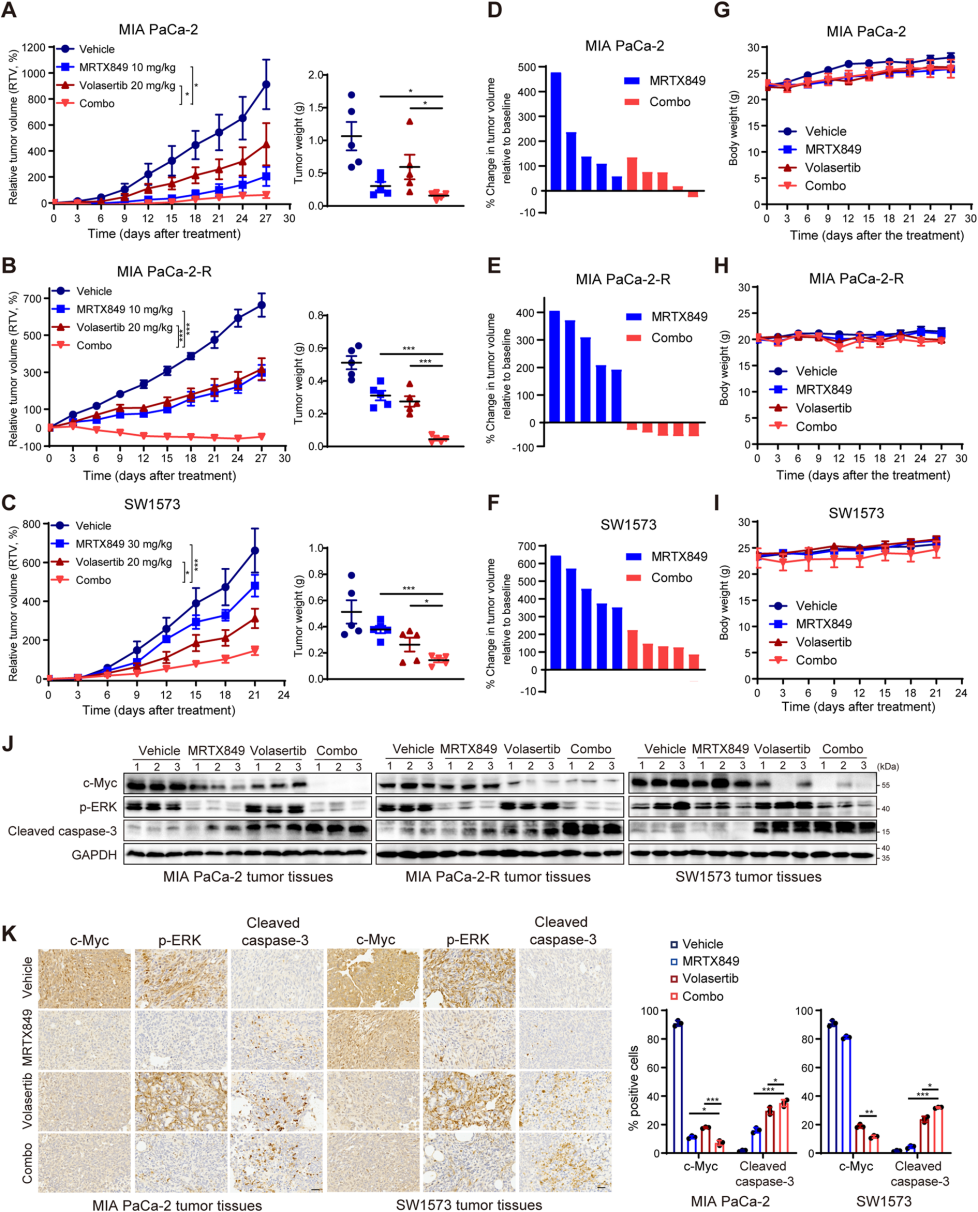
Co-inhibition of PLK1 and KRAS^G12C^ synergistically suppresses tumor growth and overcomes resistance in vivo. Relative tumor volumes (RTV) of KRAS^G12C^-mutant MIA PaCa-2 (**A**), MIA PaCa-2-R (**B**) and SW1573 (**C**) cell xenografts treated with vehicle, MRTX849 (10 or 30 mg/kg/day), Volasertib (20 mg/kg/week), alone or in combination; statistical significance was evaluated by one-way ANOVA with Tukey’s multiple comparison test. *P < 0.05, **P < 0.01, and ***P < 0.001. Data are the mean ± SEM (5 mice/group). Tumors in each model were weighted at the end of the treatments. **D**–**F** Changes in tumor volumes of individual mice in MIA PaCa‑2 (**D**), MIA PaCa‑2-R (**E**) and SW1573 (**F**) xenografts at the end of treatment were calculated. **G**–**I** Mouse body weights in each model were measured every 3 days during the experiments. **J** Western blot analyses of the tumor tissues from the above xenografts. **K** IHC analyses of MIA PaCa-2 and SW1573 tumor tissues after the treatment. Scale bar, 50 μm. Cells positive for c-Myc and cleaved caspase-3 were quantified (n = 3). Data are shown as mean ± SD. Statistical significance was assessed using two-tailed unpaired Student’s t test. *P < 0.05, **P < 0.01, and ***P < 0.001

Moreover, we found that the combination of KRAS^G12C^i and PLK1i inhibited both RAS/MAPK and PLK1 signaling pathways, as shown by suppressed activation of ERK and c-Myc. Cell cycle blockade in G2/M phase was associated with elevated levels of Cyclin B1 and p-Histone H3, and reduced amounts of cell division control protein 25C (cdc25C) [39]. Additionally, the combination significantly induced cell apoptosis, as evidenced by strong detection of caspase-3 cleavage (Fig. 3I and Additional file 2: Fig. S4E). Thus, KRAS^G12C^i in combination with PLK1i exhibited synergistic cytotoxic effects and abrogate resistance in KRAS^G12C^-mutant cancer cells by concomitant suppression of both ERK-dependent and -independent c-Myc expression.

### Co-inhibition of PLK1 and KRAS^G12C^ synergistically suppresses tumor growth and overcomes resistance in vivo

Building on these promising cellular data, we further assessed whether targeting both the mitotic machinery and c-Myc via PLK1i led to the enhanced antitumor activity of KRAS^G12C^i in vivo. We co-administered Volasertib with MRTX849 in three KRAS^G12C^-mutant tumor models: KRAS^G12C^i-sensitive MIA PaCa-2, -intrinsic resistant SW1573 and -acquired resistant MIA PaCa-2-R cells. In agreement with the in vitro sensitivity, MRTX849 monotherapy led to a more pronounced antitumor effect in the MIA PaCa-2 xenotransplanted model compared with that in the MIA PaCa-2-R and SW1573 xenograft models (Fig. 4A, C). Volasertib alone exhibited moderate tumor inhibition in these mouse models. However, KRAS^G12C^i co-administered with Volasertib showed a statistically greater efficacy compared to each agent alone in all the tested xenograft models (Fig. 4A–F). Importantly, co-treatment led to overwhelming tumor regressions in MIA PaCa-2-R model, with a 100% partial tumor regression (PR) of all mice (Fig. 4E). Visual inspection at the experimental endpoint revealed consistently lighter tumor weights for the MRTX849 and Volasertib cohort in comparison with all single-drug treatments, with no evidence of drug-induced body weight loss (Fig. 4G, I and Additional file 2: Fig. S4F). Overall, PLK1 blockage enhanced KRAS^G12C^i efficacy in KRAS^G12C^-mutant tumors, delayed cancer progression and counteracted drug resistance.

Finally, both immunoblotting and immunostaining results revealed that MRTX849 monotherapy inhibited ERK phosphorylation in all the aforementioned mouse models, but no obvious decrease of c-Myc in the KRAS^G12C^i-resistant xenograft tumors (Fig. 4J, K), indicating that the alteration of c-Myc level could be helpful for predicting tumor responses to KRAS^G12C^i treatment. Whereas Volasertib-mediated antitumor activity indistinguishably correlated with a pharmacodynamic reduction of c-Myc and an induction of caspase-3 cleavage, whose effects were largely augmented under the co-administration with MRTX849 (Fig. 4J, K). These suggested that PLK1i in combination with KRAS^G12C^i demonstrated a great cooperativity in vivo, which might represent a promising therapeutic strategy.

### ST8SIA6-AS1 promotes malignant proliferation of KRAS^G12C^-mutant cancers through aurora A/PLK1/c-Myc activation

**Fig. 5:**
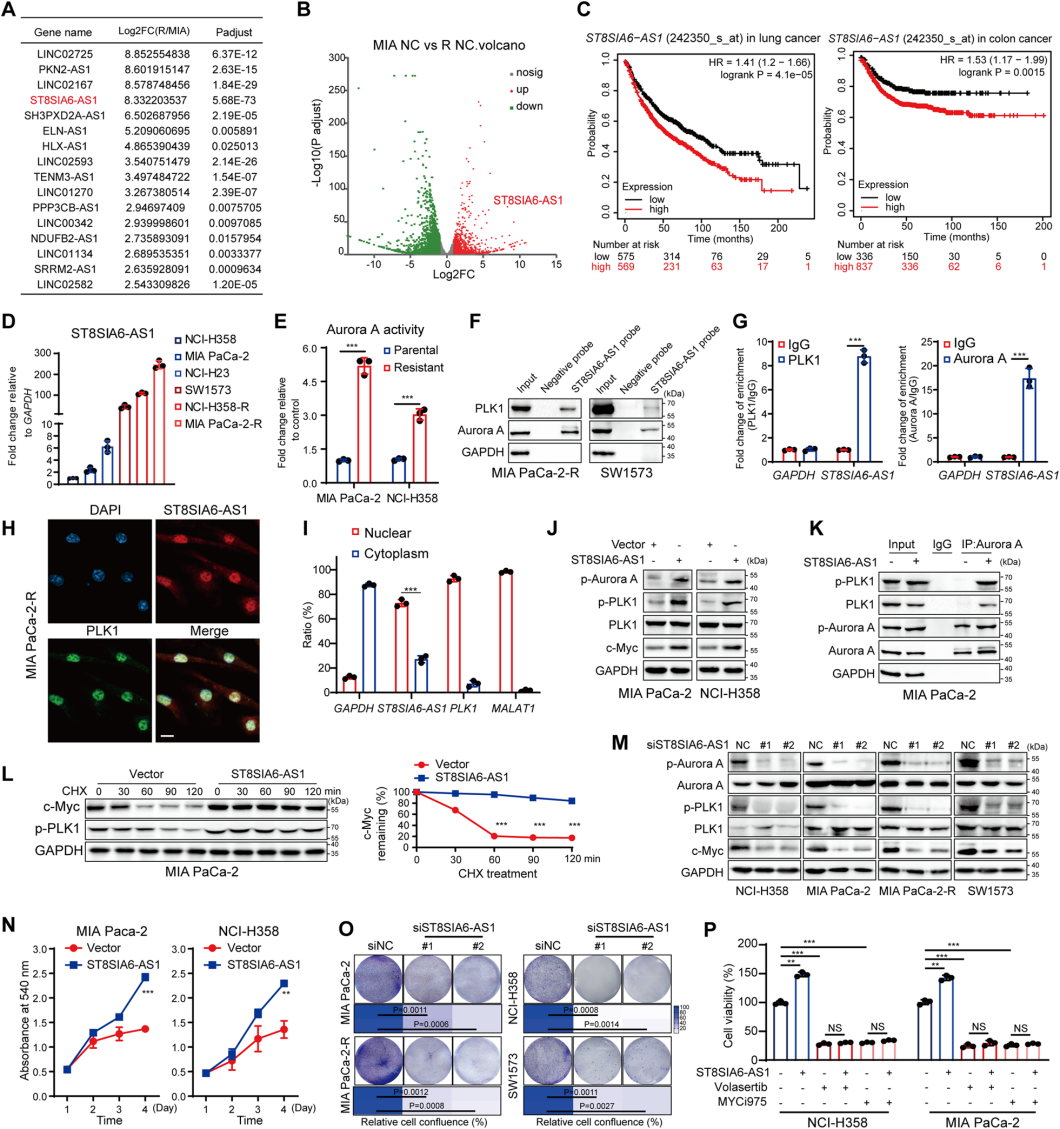
ST8SIA6-AS1 promotes malignant proliferation of KRAS^G12C^-mutant cancers through Aurora A/PLK1/c-Myc activation. **A** A table displayed the top 16 most increased lncRNAs in MIA PaCa-2-R cells compared to parental cells. **B** The volcano plot of the DEGs showing the significantly up-regulated ST8SIA6-AS1. **C** Association of ST8SIA6-AS1 expression with overall survival (OS) in all lung cancer patients (n = 1880) or colon cancer (n = 1302) patients from TCGA database. **D** RT-qPCR of ST8SIA6-AS1 expression in six KRAS^G12C^-mutant cell lines. **E** The relative Aurora A phosphorylation levels in MIA PaCa-2 or NCI-H358 parental and resistant cells were tested by western blotting and quantified. **F** RNA pull-down by full-length biotin-labeled ST8SIA6-AS1 was then subjected to immunoblotting with PLK1 and Aurora A. **G** RT-qPCR of ST8SIA6-AS1 retrieved by RNA immunoprecipitation (RIP) with PLK1 or Aurora A antibodies in MIA PaCa-2-R cells. **H** The co-localization of ST8SIA6-AS1 and PLK1 in MIA PaCa-2-R cells was detected by confocal microscopy. Scale bar, 10 μm. **I** RT-qPCR showing the nuclear and cytoplasmic fraction of ST8SIA6-AS1 in MIA PaCa-2-R cells, with *GAPDH* and *MALAT1* as cytoplasmic and nuclear control, respectively. **J** Western blot analysis of phosphorylated PLK1 and Aurora A levels were determined in cells transfected with plasmid pcDNA3.1-ST8SIA6-AS1. **K** Co-IP with Aurora A antibody in ST8SIA6-AS1-overexpressed cells. **L** Western blotting of c-Myc in vector- or ST8SIA6-AS1- expressing MIA PaCa-2 cells treated with CHX. The quantification of c-Myc degradation rate by gray scale analysis was shown. **M** Western blot analysis of phosphorylated PLK1 and Aurora A levels in ST8SIA6-AS1-depleted cells. **N** The proliferation of ST8SIA6-AS1-overexpressed cells was determined by SRB assay. **O** Colony formation assay of ST8SIA6-AS1 knockdown cells. **P** Relative cell viability was tested in ST8SIA6-AS1-overexpressed NCI-H358 and MIA PaCa-2 cells under the treatment of 5 nM Volasertib or 6 µM MYCi975 for 72 h. Data represent the average and SD. Statistical significance was assessed using two-tailed unpaired Student’s t test. *P < 0.05, **P < 0.01, ***P < 0.001. See also Additional file 2: Fig. S5

To explore the molecular mechanism underlying the activation of PLK1 in KRAS^G12C^i-resistant cells, we dug into the RNA-seq data and particularly noted that many lncRNAs were significantly differentially expressed in MIA PaCa-2-R cells compared to that in the parental ones (Additional file 2: Fig. S5A). The top-scored lncRNAs related with KRAS^G12C^i resistance, with a log2 fold-change above 2.5 were depicted in the table, among which ST8SIA6-AS1 stood out as the most significantly differentially expressed (Fig. 5A, B). ST8SIA6-AS1, also termed APAL (Aurora A/PLK1-associated lncRNA), has been revealed to harbor both hairpins that specifically interact with PLK1 and Aurora A, thereby promoting efficient phosphorylation of PLK1 by Aurora A [40]. In PAAD, COAD, LUAD and LUSC patients, ST8SIA6-AS1 expression levels were elevated in tumor tissues than corresponding normal tissues (Additional file 2: Fig. S5B). The Kaplan–Meier survival curve demonstrated a close correlation between ST8SIA6-AS1 high expression and poor overall survival of patients with lung cancer or colon cancer (Fig. 5C). Thus, we first validated that *ST8SIA6-AS1* transcripts were immensely increased in KRAS^G12C^i-resistant cell lines, whereas under-expressed in various KRAS^G12C^i-sensitive ones, including NCI-H358, MIA PaCa-2, and NCI-H23 (Fig. 5D), indicating that ST8SIA6-AS1 might be considered as a potential biomarker of KRAS^G12C^i sensitivity. Then, we verified that Aurora A was also highly-activated in KRAS^G12C^i-resistant cells and maintained upon KRAS^G12C^i treatment (Fig. 5E and Additional file 2: Fig. S5C). RNA pulldown with biotin-labeled ST8SIA6-AS1 followed by immunoblotting confirmed the binding of ST8SIA6-AS1 with PLK1 and Aurora A (Fig. 5F). RNA immunoprecipitation in MIA PaCa-2-R cells using PLK1 or Aurora A antibodies revealed profound enrichment of ST8SIA6-AS1 (Fig. 5G). Moreover, fluorescent in situ hybridization (FISH) using biotin-labelled ST8SIA6-AS1 probes followed by PLK1 immunostaining showed that ST8SIA6-AS1 and PLK1 mainly co-localized in and around the nucleus of MIA PaCa-2-R cells (Fig. 5H). And RT-qPCR of the nuclear and cytoplasmic fractionations of MIA PaCa-2-R cells also confirmed that the majority of ST8SIA6-AS1 localized in the nucleus (Fig. 5I).

Next, to validate the regulatory role of Aurora A/PLK1 activation by ST8SIA6-AS1, ST8SIA6-AS1 overexpression in MIA PaCa-2 and NCI-H358 cells by transfection with a transient expression vector were verified by RT-qPCR (Additional file 2: Fig. S5D) and seen as markedly elevated phosphorylation levels of Aurora A at Thr288 and PLK1 at Thr210, together with c-Myc upregulation (Fig. 5J). Forced expression of ST8SIA6-AS1 markedly elevated PLK1 phosphorylation and its binding with Aurora A in MIA PaCa-2 (Fig. 5K), suggesting that ST8SIA6-AS1 induced PLK1 phosphorylation through promoting Aurora A activity. Moreover, by promoting PLK1 hyper-activation, ST8SIA6-AS1 overexpression significantly increased the c-Myc stability and extended the half-life of c-Myc protein from < 1 h to > 2 h under CHX treatment (Fig. 5L). On the contrary, ST8SIA6-AS1 knockdown using two independent siRNAs significantly inhibited Aurora A and PLK1 phosphorylation in all the tested cell lines, leading to downregulation of c-Myc levels (Fig. 5M; Additional file 2: Fig. S5E).

To assess the functional role of ST8SIA6-AS1 in KRAS^G12C^-mutant cells, we tested whether modulation of ST8SIA6-AS1 level would affect cell proliferation. ST8SIA6-AS1 overexpression significantly accelerated the proliferation of KRAS^G12C^i-sensitive MIA PaCa-2 and NCI-H358 cells (Fig. 5N). While ST8SIA6-AS1 deletion tremendously reduced the clonogenicity of all the tested KRAS^G12C^i-sensitive and -resistant cell lines (Fig. 5O). Loss of ST8SIA6-AS1 caused severe mitotic abnormalities and triggered G2/M cell cycle arrest in cancer cells (Additional file 2: Fig. S5F), leading to apoptotic cell death (Additional file 2: Fig. S5G, H). Thus, ST8SIA6-AS1 played a vital role for the survival of KRAS^G12C^-mutant cancers. Furthermore, we observed that cells with forced expression of ST8SIA6-AS1 exhibited similar sensitivity to Volasertib or MYCi975 treatment compared to control cells (Fig. 5P), indicating that PLK1/c-Myc function largely mediated the tumor-promoting effect of ST8SIA6-AS1. Together, these results suggested that ST8SIA6-AS1 facilitates KRAS^G12C^-mutant cancer progression, which is primarily dependent on Aurora A/PLK1/c-Myc activation.

### Targeting ST8SIA6-AS1 reverses resistance to KRAS^G12C^i in vitro and in vivo

**Fig. 6:**
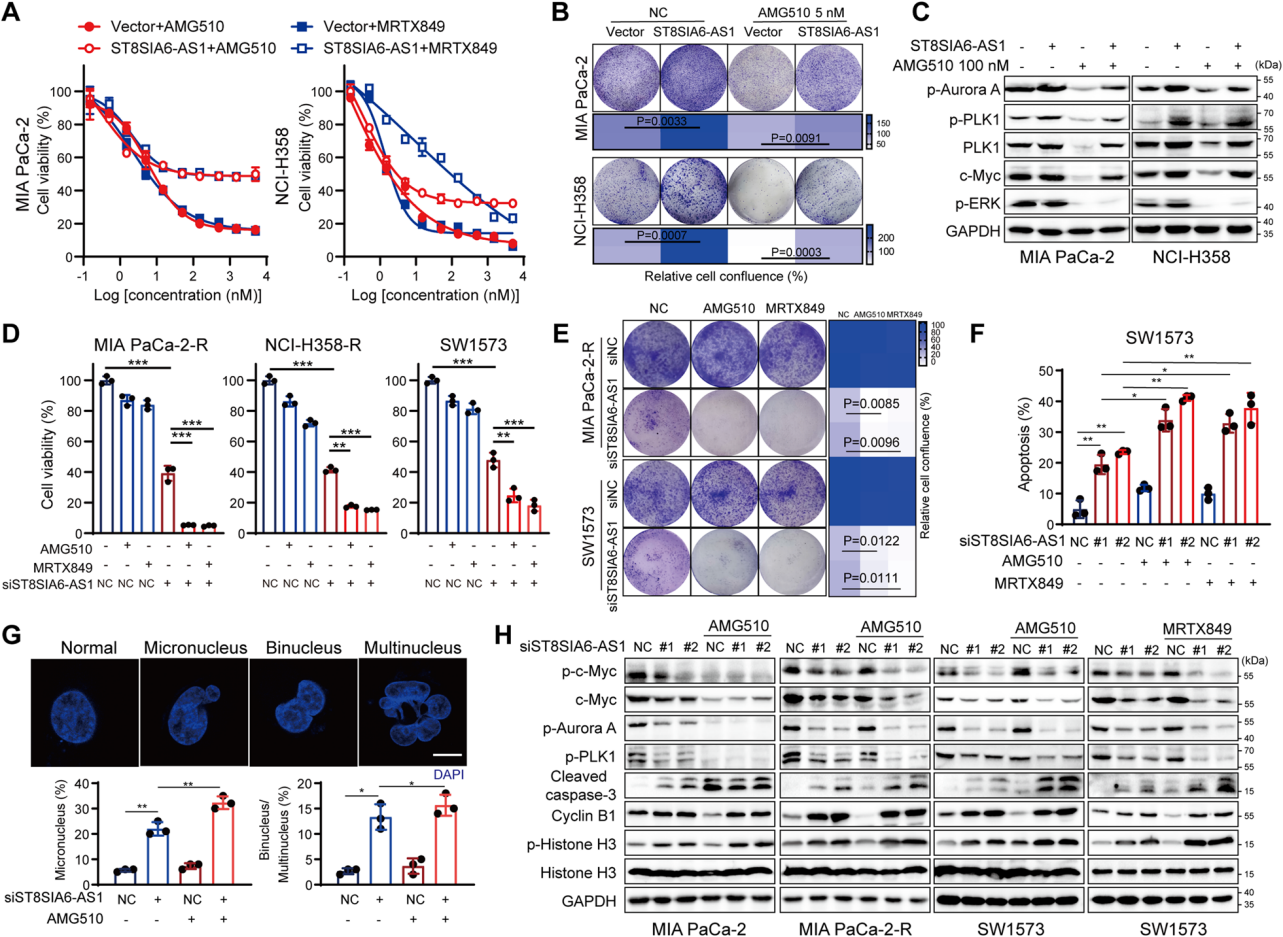
Targeting ST8SIA6-AS1 reverses cell resistance to KRAS^G12C^i in vitro. **A** Relative cell viability of ST8SIA6-AS1-overexpressed cells under increasing concentrations of KRAS^G12C^i for 72 h. **B** Cell colony formation of ST8SIA6-AS1-overexpressed cells treated with or without AMG510. **C** Western blot analysis of cells with ST8SIA6-AS1 forced expression treated by 100 nM AMG510 for 24 h. ST8SIA6-AS1-knockdown KRAS^G12C^i resistant cells were treated with 1 µM KRAS^G12C^i to determine the effect on viability by the SRB assay (72 h) (**D**) and colony formation assay (**E**). **F** Cell apoptosis analysis of ST8SIA6-AS1-knockdown SW1573 cells treated with or without 100 nM KRAS^G12C^i for 24 h. **G** The effect of ST8SIA6-AS1 depletion followed by 100 nM AMG510 treatment for 24 h on generating micronuclei and multi-nuclei. MIA PaCa-2-R cells were immunostained with DAPI. The percentages of abnormal mitotic cells were calculated. Scale bar 10 μm. **H** Immunoblotting of ST8SIA6-AS1 knockdown cells treated with 100 nM KRAS^G12C^i for 24 h. Statistical significance was assessed using two-tailed unpaired Student’s t test. *P < 0.05, **P < 0.01, ***P < 0.001. See also Additional file 2: Fig. S6

To investigate whether ST8SIA6-AS1 hyper-expression is the key factor conferring Aurora A/PLK1 constitutive activation and tolerance to KRAS^G12C^ inhibition, we determined the alteration of sensitivity to KRAS^G12C^i after forced-expression of ST8SIA6-AS1 in KRAS^G12C^i-sensitive cell lines, MIA PaCa-2 and NCI-H358, which exhibited low copy numbers of ST8SIA6-AS1. We observed that MIA PaCa-2 and NCI-H358 cells overexpressing ST8SIA6-AS1 became resistant to KRAS^G12C^i compared to control cells (Fig. 6A, B and Additional file 2: Fig. S6A). These ST8SIA6-AS1-overexpressed cells exhibited elevated Aurora A/PLK1 activation and ERK-independent c-Myc persistence under AMG510 treatment (Fig. 6C). Whereas ST8SIA6-AS1 depletion in MIA PaCa-2-R, NCI-H358-R and SW1573 cells restored cell sensitivity to KRAS^G12C^i (Fig. 6D, E). Of note, knockdown of ST8SIA6-AS1 in SW1573 cells followed by KRAS^G12C^i treatment showed higher percentages of cell apoptosis (Fig. 6F and Additional file 2: Fig. S6B). In addition, a portion of the ST8SIA6-AS1-depleted MIA PaCa-2-R cells exited mitosis before undergoing cell death, forming giant cells with micronucleation (Fig. 6G), which defines mitotic errors [41], as well as binucleation and multinucleation, manifesting cells that would eventually undergo mitotic catastrophe [42]. And the combined treatment with KRAS^G12C^i exhibited more severe mitotic abnormalities and mitotic catastrophes, leading to G2/M cell cycle arrest and massive cell death (Fig. 6G and Additional file 2: Fig. S6C). Western blot analysis revealed that the combination led to Aurora A/PLK1 inhibition and c-Myc reduction, meanwhile inducing histone H3 (S10) phosphorylation and caspase-3 cleavage (Fig. 6H). These results demonstrated that hyper-expression of ST8SIA6-AS1 in KRAS^G12C^i-resistant cells persistently activates Aurora A/PLK1/c-Myc signaling and confers resistance to KRAS^G12C^i.

**Fig. 7:**
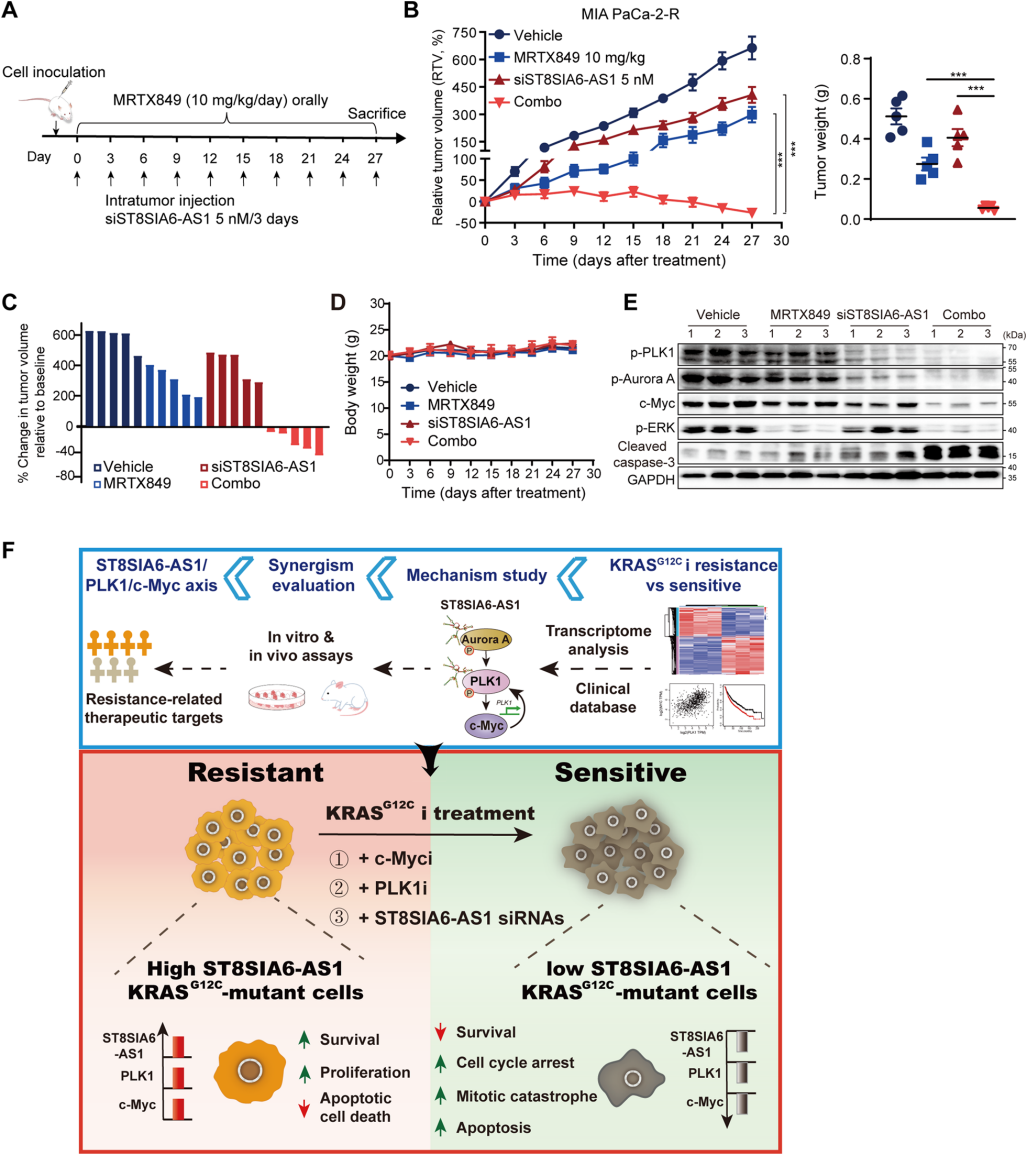
Suppression of ST8SIA6-AS1 restores the sensitivity of KRAS^G12C^i in vivo. **A** MIA PaCa-2-R cells were inoculated to form xenografts in nude mice (n = 5 mice/group). The administration dose and time of the drugs were recorded. **B** RTV of MIA PaCa-2-R xenografts treated with vehicle, MRTX849 and ST8SIA6-AS1 siRNA, alone or in combination. Tumor weights were measured after surgical resection. Data are the mean ± SEM (5 mice/group). *P < 0.05, **P < 0.01, and ***P < 0.001 by one-way ANOVA with Tukey’s multiple comparison test. **C** Changes in tumor volumes of individual mice in MIA PaCa‑2-R xenografts at the end of treatment. **D** Mouse body weights in MIA PaCa-2-R xenograft model were measured every 3 days during the experiments. Data are the mean ± SD. Statistical significance was assessed using two-tailed unpaired Student’s t test. *P < 0.05, **P < 0.01, ***P < 0.001. **E** Western blot analysis of tumor tissues at the end of the treatment. See also Additional file 2: Fig. S6. **F** Schematic model depicting the mechanism by which concurrent targeting of ST8SIA6-AS1/PLK1/c-Myc axis with KRAS elicits significant KRAS^G12C^i-resistant cell death

Recent preclinical studies suggest that RNA interference technologies including siRNAs, ASOs (antisense oligonucleotides) and LNAs (Locked nucleic acid) have been applied to inhibit target transcripts and hold promise for the treatment of various cancers [43, 44, 45]. Subsequently, we evaluated the therapeutic efficacy of ST8SIA6-AS1 siRNA treatment in combination with KRAS^G12C^i using MIA PaCa-2-R cell xenograft in nude mice (Fig. 7A). Significant regressions of tumor growth (100% PR) were detected in the combinational group compared to single-drug groups (Fig. 7B, C and Additional file 2: Fig. S6D). Moreover, both ST8SIA6-AS1 siRNA and the combinational treatment were deemed safe as indicated by no significant change in mouse body weights (Fig. 7D). As expected, tumor tissues from these animals showed decreased levels of ST8SIA6-AS1 expression (Additional file 2: Fig. S6E) and suppressed Aurora A/PLK1 signaling activation (Fig. 7E). Severe cell death was visualized by marked c-Myc degradation and caspase-3 cleavage (Fig. 7E), indicating that ST8SIA6-AS1 depletion successfully restored KRAS^G12^i sensitivity and cooperatively caused tumor regressions in resistant xenograft model in vivo.

## Discussion

Although many patients treated with KRAS^G12C^i have clinical benefits, due to genetic heterogeneity and various compensation mechanisms, most patients eventually develop acquired resistance to single-agent therapy. Notably, the sensitivity and resistance to KRAS^G12C^i were not predicted by KRAS allele zygosity status [3]. In our study, we explored the potential mechanisms that mediate both intrinsic and acquired resistance to KRAS^G12C^i in KRAS^G12C^-mutant cancers. We identified a reciprocal alteration of c-Myc expression as an indicator of cell sensitivity to KRAS^G12C^i. KRAS^G12C^i reduced c-Myc levels in responsive cells in a time- and dose-dependent manner. While cells with acquired resistance to KRAS^G12C^i, MIA PaCa-2-R, NCI-H358-R and Ba/F3 KRAS^G12C^-R, or SW1573 cells with intrinsic resistance showed sustained c-Myc levels during drug exposure, which were also recapitulated in xenograft models. Combined inhibition of c-Myc and KRAS was effective to abrogate KRAS^G12C^i resistance and potently enhanced the drug efficacy in NSCLC and PAAD cells with KRAS^G12C^ mutation. Whereas currently there are few c-Myc inhibitors showing promising outcome in clinical trials [46]. Therefore, intense efforts are being devoted to developing more specific and effective c-Myc targeting strategies.

Early studies have shown that RAS stimulates the RAF/MEK/ERK signaling activation, resulting in ERK-dependent c-Myc phosphorylation at S62 and increased c-Myc protein stability [47, 48]. However, we noticed that resistant cells exhibited basal phosphorylation of ERK compared to the parental ones. Administration of KRAS^G12C^i induced significantly different anti-proliferative effects on the parental and resistant cells despite nearly the same degree of ERK suppression, suggesting that the inhibition of MAPK pathway was not an ideal indicator of KRAS^G12C^i sensitivity. Thus, both de novo and acquired tolerant cells exhibited ERK-dependent and -independent mechanisms to maintain c-Myc protein levels. Through transcriptome and protein phosphorylation analyses, we proposed a model whereby KRAS^G12C^i resistance presents ST8SIA6-AS1-dependent feed-forward activation of Aurora A/PLK1, leading to c-Myc phosphorylation and stabilization. In accordance with the previous finding that KRAS^G12C^i resistance was accompanied by MYC overexpression in clinical practice [8], we further expounded the resistance-monitoring role of c-Myc and illustrated the intricate mechanism accounting for its elevation. Of course, other compensatory pathways including PI3K/AKT/mTOR [47], MEK5/ERK5 [30] and GSK-3α/TAK1/Table [49] may also contribute to c-Myc maintenance in KRAS^G12C^i-resistant cells under KRAS^G12C^i treatment. We suspected that the kinase networks manipulating c-Myc after KRAS inhibition are considerably more complicated than currently understood.

Preclinical studies showed that the stimulation of proliferative signaling was one of the dominant features of KRAS^G12C^i resistance [50]. Our integrative transcriptional analysis revealed a highly enriched and significantly upregulated G2/M cell cycle-related gene set, notably *PLK4* and *PLK1* in the resistant cells. As previously reported [51, 52], KRAS^G12C^i-resistant cells were sensitive to Aurora kinase inhibitors. Whereas we uncovered that this vulnerability extends to other spindle assembly regulators like PLK1, which, on the other hand, is also a powerful regulator of c-Myc activity. PLK1 is overexpressed in a broad spectrum of human cancers and correlates with unfavorable patients’ outcomes [53, 54]. PLK1 inhibitors have been widely developed in preclinical studies and clinical trials for NSCLC and PAAD. Despite the limited efficacy of phase II trials in NSCLC patients [55], PLK1 is considered a synthetic lethal target in KRAS-mutant cancers, which are proved to be sensitive to mitotic perturbations and hypersensitive to the PLK1 suppression [37]. Whereas the crosstalk between PLK1 and oncogenic KRAS signaling remains incompletely elucidated and the therapeutic effect of co-inhibition is worth evaluating. Our finding revealed a reciprocal regulation between PLK1 and the KRAS downstream effector c-Myc. PLK1 mediates c-Myc S62 phosphorylation and enhances its stability, which in return promotes PLK1 transcription. Pharmacologic inhibition of PLK1 reduced c-Myc expression, arrested cells in G2/M phase, induced mitotic errors and triggered apoptotic cell death, and these effects were augmented when combined with KRAS^G12C^i. Co-administration of Volasertib with MRTX849 was able to dampen tumor progression in all the tested sensitive and resistant cell xenograft models, accompanied with synergistic loss of c-Myc. Furthermore, PLK1i alone could markedly inhibit the proliferation of KRAS^G12C^-expressing Ba/F3 cells harboring on-target second-site mutations, known as common mutations contributing to drug resistance in clinic. More importantly, both PLK1i and MRTX849 have been reported to exhibit great blood-brain barrier permeability [56–58]. Brain metastasis occurs in about 20–40% of malignant tumors, such as lung cancer, breast cancer, and melanoma, acknowledged as a devastating complication with poor outcomes. In particular, lung cancer accounts for approximately 50% of all brain metastases and poses a considerable therapeutic challenge [59]. Thus, we speculated that KRAS^G12C^ cancer patients with brain metastasis may be acutely susceptible to the combination of PLK1i and MRTX849, which may show immediate clinical therapeutic significance. Overall, we suggested that PLK1 inhibition is a potent strategy to restrain c-Myc and overcome resistance to KRAS^G12C^i. Our data also supported the notion that the KRAS-mutant cells are quite sensitive to the concurrent targeting of KRAS and specific cell cycle-related participants, including c-Myc, CDKs, PLKs, and Aurora kinases, whose inhibitors currently exhibit limited single-agent efficacy in clinical trials [60–62].

We also confirmed the limitations of KRAS^G12C^i monotherapy and indicated that the abundance of lncRNAs, including ST8SIA6-AS1 we investigated in our research, may help to predict the therapeutic outcome of KRAS^G12C^i in clinical trials. ST8SIA6-AS1, also known as APAL, has been identified to specifically and directly interact with Aurora A and PLK1, facilitating PLK1 phosphorylation and activation [41]. It has been shown that ST8SIA6-AS1 overexpression is observed in human breast and liver cancers, and positively correlated with poor prognosis [63, 64]. Silencing of ST8SIA6-AS1 disrupted cell cycle process, tumor growth and metastasis [65–67]. We substantially extended these earlier observations in cancer scope and mechanistically demonstrated the function and mechanism of ST8SIA6-AS1 in the proliferation and chemoresistance of KRAS^G12C^-mutant cancers. ST8SIA6-AS1 is characterized as a driver of both intrinsic and acquired resistance to KRAS^G12C^i via PLK1 activation and c-Myc stabilization. Its expression is more abundant in tumor tissues from PAAD, CRC and NSCLC cancer patients than reciprocally normal tissues. ST8SIA6-AS1 depletion repressed the phosphorylation and activation of PLK1 and Aurora A, leading to G2/M phase arrest, chromosome abnormality and cell apoptosis. The siRNAs targeting ST8SIA6-AS1 portrayed a more remarkable antitumor effect in the resistant mouse model when combined with KRAS^G12C^i. Therefore, when determining the treatment of cancers bearing KRAS^G12C^-mutation, the evaluation of ST8SIA6-AS1 level is advised to select patients who will be more likely to benefit from KRAS^G12C^i therapy.

Through lncRNA library screening based on RNA-seq, we identified ST8SIA6-AS1 as one of the candidate genes involved in KRAS^G12C^i resistance. Although we focused on ST8SIA6-AS1, there are also other lncRNAs of interest on the candidate list that deserve to be explored in future studies. In addition, we have not clarified why ST8SIA6-AS1 expression is significantly amplified in the KRAS^G12C^i-resistant cells. It showed that ST8SIA6-AS1 transcripts were only modestly induced by KRAS^G12C^i (Additional file 2: Fig. S6F). We speculate that it may serve as an adaptive factor in response to KRAS signaling inhibition, whose abundance accumulates over treatment time, ultimately driving strong activation of PLK1/c-Myc signaling, leading to long-term resistance. The exact mechanism requires further investigations. Moreover, it’s acknowledged that epithelial-to-mesenchymal transition (EMT) has been identified as an important cause to KRAS^G12C^i resistance [68]. Due to its stimulative role on tumor EMT and metastasis [67, 69], high levels of ST8SIA6-AS1 may dampen the efficacy of KRAS^G12C^i in KRAS^G12C^-mutant cancers through EMT induction. In consequence, to decipher the specific functions of ST8SIA6-AS1 in different types of human cancer, identifying its unique interacting molecules will be critical.

## Conclusion

In summary, we illuminated that upregulation of lncRNA ST8SIA6-AS1 transcripts enables hyper-activation of the Aurora A/PLK1/c-Myc pathway, leading to both intrinsic and acquired resistance to KRAS^G12C^i. By identifying this pathway as a susceptibility of KRAS^G12C^i-resistant tumors, we recommended combinational strategies that could be exploited in KRAS^G12C^-mutated cancer patients and indicated the probabilities of high abundance of c-Myc or ST8SIA6-AS1 as clinical biomarkers during therapy. Given the tremendous promise of RNA-based anti-cancer therapy, and the fact that both KRAS^G12C^i and PLK1i are currently under clinical development, we put forward the idea that concurrent targeting of KRAS with ST8SIA6-AS1 or PLK1 in KRAS^G12C^i-resistant patients is an open and prospective opportunity.

### Supplementary Information

**Additional file 1: Table S1.** Cell lines and culture conditions. **Table S2.** Reagents and tools table. **Table S3.** siRNA sequences in RNA interference. **Table S4.** Sequences in PCR, RT-qPCR and CHIP-qPCR.

**Additional file 2: Figure S1.** C-Myc alteration predicts for the response to KRAS^G12C^i. **Figure S2.** C-Myc is vital for KRAS^G12C^-mutant cell proliferation and mediates resistance to KRAS^G12C^i. **Figure S3.** The reciprocal activation of PLK1/c-Myc pathway confers resistance to KRAS^G12C^i. **Figure S4.** PLK1i synergizes with KRAS^G12C^i via ERK-dependent and -independent downregulation of c-Myc. **Figure S5.** ST8SIA6-AS1 promotes malignant proliferation of KRAS^G12C^-mutant cancers through Aurora A/PLK1/c-Myc activation. **Figure S6.** Targeting ST8SIA6-AS1 reverses cell resistance to KRAS^G12C^i.

## Data Availability

All data generated or analyzed during this study are included in this published article (and its Additional files) and available from the corresponding author on reasonable request.

## Abbreviations

ANOVA: Analysis of variance
ASOs: Antisense oligonucleotides
ATCC: American type culture collection
Aurora A: Aurora kinase A
ChIP: Chromatin immunoprecipitation
CHX: Cycloheximide
COAD: Colon adenocarcinoma
CRC: Colorectal cancer
DAB: Diaminobenzidine
DAPI: 4′,6-Diamidino-2-phenylindole
Dox: Doxycycline
GSEA: Gene set enrichment analysis
IC_50_s: The half-maximal inhibitory concentrations
IHC: Immunohistochemistry
KEGG: Kyoto encyclopedia of genes and genomes
LNAs: Locked nucleic acid
LncRNA: Long non-coding RNA
LUAD: Lung adenocarcinoma
LUSC: Lung squamous cell carcinoma
MTT: Methyl thiazolyl tetrazolium
NSCLC: Nonsmall cell lung cancer
OS: Overall survival
PAAD: Pancreatic adenocarcinoma
PI: Propidium iodide
PLK1: Polo-like kinase 1
PR: Partial regression
RIP: RNA immunoprecipitation
RNA FISH: RNA fluorescence in situ hybridization
RNA-seq: RNA sequencing
RT-qPCR: RNA extraction and quantitative real-time PCR
SD: Standard deviation
SEM: Standard error of the mean
SRB: Sulforhodamine B
STR: Short tandem repeat
TCGA: The Cancer Genome Atlas
TSS: Transcription start site
