# Supplementary material for: Targeting ST8SIA6-AS1 counteracts KRASG12C inhibitor resistance through abolishing the reciprocal activation of PLK1/c-Myc signaling
Source: Exp Hematol Oncol. 2023 Dec 16;12:105. doi: 10.1186/s40164-023-00466-3 (PMC10724920; doi:10.1186/s40164-023-00466-3)
Supplement: Supplementary file 1 — Additional file 1: Table S1. Cell lines and culture conditions. Table S2. Reagents and tools table. Table S3. siRNA sequences in RNA interference. Table S4. Sequences in PCR, RT-qPCR and CHIP-qPCR. [file 40164_2023_466_MOESM1_ESM.pdf]

Table S1. Cell lines and culture conditions.

Cell lines	Culture condition	Source	Identifier
MIA PaCa-2	Dulbecco's Modified Eagle's Medium + 10%FBS + 2.5% horse serum	ATCC (Manassas, VA, USA)	CRM-CRL-1420
NCI-H358	RPMI-1640 + 10%FBS	ATCC	CRL-5807
NCI-H23	RPMI-1640 + 10%FBS	ATCC	CRL-5800
NCI-H1373	RPMI-1640 + 10%FBS	ATCC	CRL-5866
SW1463	Leibovitz's L-15+ 10%FBS	ATCC	CCL-234
SW1573	Leibovitz's L-15+ 10%FBS	ATCC	CRL-2170
293T	Dulbecco's Modified Eagle's Medium + 10%FBS	ATCC	CRL-3216
Ba/F3	RPMI-1640 + 10%FBS	German Collection of Microorganisms and Cell Cultures (DSMZ)	ACC 300

Table S2. Reagents and Tools table.

**Antibodies and reagents**	**Source**	**Identifier**
Antibodies		
Rabbit polyclonal anti-pThr202/pTyr204 ERK1/2	Cell Signaling Technology (CST)	Cat#: 9010; RRID: AB_331646
Rabbit monoclonal anti-ERK1/2	CST	Cat#: 4695; RRID: AB_390779
Rabbit monoclonal anti-p-MEK1/2 (Ser217/221)	CST	Cat#: 9154; RRID: AB_2138017
Mouse monoclonal anti-MEK1/2	CST	Cat#: 4694; RRID: AB_10695868
Rabbit monoclonal anti-p-PLK1 (Thr210)	CST	Cat#: 9062; RRID: AB_11127447
Rabbit monoclonal anti-PLK1	CST	Cat#: 4513; RRID: AB_2167409
Rabbit monoclonal anti-p-Aurora A (Thr288)	CST	Cat#: 3079; RRID: AB_2061481
Rabbit monoclonal anti-Aurora A	CST	Cat#: 91590; RRID: AB_2800171
Rabbit monoclonal anti-p-c-Myc (Ser62)	CST	Cat#: 13748; RRID: AB_2687518
Rabbit monoclonal anti-c-Myc (WB)	CST	Cat#: 18583; RRID: AB_2895543
Rabbit monoclonal anti-c-Myc (IHC)	Abcam	Cat#: ab32072; RRID: AB_731658
Rabbit monoclonal anti-Cleaved caspase-3	CST	Cat#: 9664; RRID: AB_2070042
Rabbit monoclonal anti-p-Histone H3 (Ser10)	CST	Cat#: 9706; RRID: AB_331748
Rabbit monoclonal anti-Histone H3	CST	Cat#: 4499; RRID: AB_10544537
Rabbit monoclonal anti-cdc25C	CST	Cat#: 4688; RRID: AB_560956
Rabbit monoclonal anti-Cyclin B1	CST	Cat#: 12231; RRID: AB_2783553
Rabbit monoclonal anti-CDK2	CST	Cat#: 18048; RRID: AB_2923174
Rabbit monoclonal anti-Cyclin D1	CST	Cat#: 55506; RRID: AB_2827374
Rabbit monoclonal anti-GAPDH	CST	Cat#: 5174; RRID: AB_10622025
Goat anti-Mouse IgG (H+L) Highly Cross-Adsorbed Secondary Antibody, Alexa Fluor™ 594	Thermo Fisher Scientific	Cat#: A-11032; RRID:
Goat anti-Rabbit IgG (H+L) Cross-Adsorbed Secondary Antibody, Alexa Fluor™ 488	Thermo Fisher Scientific	Cat#: A-11008; RRID:
**Chemicals, peptides, and recombinant proteins**		
AMG510	MedChemExpress	Cat#: HY-114277
MRTX849	MedChemExpress	Cat#: HY-130149
MYCi975	Selleck	Cat#: S8906
Volasertib	MedChemExpress	Cat#: HY-12137
BI 2536	MedChemExpress	Cat#: HY-50698
Cycloheximide (CHX)	MedChemExpress	Cat#: HY-12320
MG132	MedChemExpress	Cat#: HY-13259
**Reagents**		
Fetal bovine serum (FBS, Gibco)	Thermo Fisher Scientific	Cat#:10099141
Non-essential amino acids (NEAA)	Thermo Fisher Scientific	Cat#:11140050

Sodium pyruvate	Thermo Fisher Scientific	Cat#:11360070
4',6-diamidino-2-phenylindol (DAPI)	Thermo Fisher Scientific	Cat#: D1306
Lipofectamine RNAiMAX	Thermo Fisher Scientific	Cat#: 13778150
Lipofectamine 2000	Thermo Fisher Scientific	Cat#: 11668019
TurboFectin 8.0	OriGene	Cat#: TF81001
RIPA lysis buffer	Thermo Fisher Scientific	Cat#: 89900
Dimethylsulfoxide (DMSO)	Sigma-Aldrich	Cat#: BML-KI597-0400
Thiazolyl Blue (MTT)	Sigma-Aldrich	Cat#: M5655
Dithiothreitol (DTT)	Sigma-Aldrich	Cat#: 43815
Sulforhodamine B (SRB)	Sigma-Aldrich	Cat#: 341738
Tween 80	Sigma-Aldrich	Cat#: P4780
Triton-X100	Sigma-Aldrich	Cat#: X100
Bovine serum albumin (BSA)	Sigma-Aldrich	Cat#: B2064
Normal goat serum	JACKSON	Cat#: 005-000-121
Normal Rabbit IgG	CST	Cat#: 2729S
Goat anti-Rabbit IgG (H+L) Secondary Antibody, HRP	Invitrogen	Cat#: 65-6120
Protease inhibitor cocktail	Roche	Cat#: 04693159001
Phosphatase inhibitor cocktail	Roche	Cat#: 04906837001
RNase A	Beyotime Biotechnology	Cat#: ST576
Propidium iodide (PI)	Beyotime Biotechnology	Cat#: ST511
Phosphate buffer saline (PBS)	Beyotime Biotechnology	Cat#: C0221A
4% paraformaldehyde	Beyotime Biotechnology	Cat#: 10455491
NP-40	Beyotime Biotechnology	Cat#: ST366
Sodium dodecyl sulfate (SDS)	Biosharp	Cat#: BS028B
Proteinase K	ABclonal	Cat#: RP02503LQ
2X Saline Sodium Citrate Buffer	MACKLIN	Cat#: X861541
**Recombinant DNA**		
pCDH-puro-c-Myc	Addgene	Cat#: 46970; RRID: Addgene_46970
FUW-tetO-hMYC	Addgene	Cat#: 20723; RRID: Addgene_20723

pcDNA3-PLK1(829)	Addgene	Cat#: 39845; RRID: Addgene_39845
pRL-TK	Addgene	Cat#: E2241; RRID: Addgene_ E2241
pGL6	Beyotime	Cat#: D2102
pcDNA3.1-ST8SIA6-AS1	This paper	N/A
**Critical commercial assays**		
BCA Protein Assay Kit	Thermo Fisher Scientific	Cat#: 23227
PARIS RNA Purification Kit	Thermo Fisher Scientific	Cat#: AM1921
PrimeScript™ RT Master Mix (Perfect Real Time)	Takara	Cat#: RR036A
TB Green® Premix Ex Taq™ II (Tli RNaseH Plus)	Takara	Cat#: RR820B
Annexin V-FITC/PI double staining apoptosis kit	BD biosciences	Cat#: 556547
RNA Labeling Mix	RiboBio Biotech	Cat#: C11002-1
EZ-Magna RIP kit	MERCK	Cat#: 17-701
SimpleChIP® Plus Enzymatic Chromatin IP Kit (Magnetic Beads)	CST	Cat#: 9005
Dual-Luciferase reporter assay system	Promega	Cat#: E1910
**Software and Algorithms**		
GraphPad Prism software 8.0	N/A	https://www.graphpad.com/
GSEA	N/A	http://software.broadinstitute.org/gsea/index.jsp
DAVID	N/A	http://david.abcc.ncifcrf.gov/
FlowJo (version 10.7.2)	BD Biosciences	https://www.flowjo.com/solutions/flowjo/downloads
Image J	Schneider et al.[1]	https://imagej.nih.gov/ij/
sgRNA design tool	Benchling	https://benchling.com
CalcuSyn software	Biosoft	https://calcusyn.software.informer.com/download/

Table S3. siRNA sequences in RNA interference

Gene	siRNA sense (5'-3')	siRNA antisense (5'-3')
c-Myc #1	GUGCAGCCGUAUUUCUACUTT	AGUAGAAAUACGGCUGCACTT
c-Myc #2	GAACACACAACGUCUUGGATT	UCCAAGACGUUGUGUGUUCTT
PLK1 #1	GGGCGGCUUUGCCAAGUGC	GCACUUGGCAAAGCCGCCC
PLK1 #2	GACAGCCUGCAGUACAUAG	CUAUGUACUGCAGGCUGUC
ST8SIA6-AS1 #1	GCUACAGAGAAGAGAGUAA	UUACUCUUCUCUGUAGC
ST8SIA6-AS1 #2	CCCAGAACCUAUUGGAACU	AGUUCCAAUAGGUUCUGGG
siNC	UUCUCCGAACGUGUCACGU	UCGUGACACGUUCGGAGAA

Table S4. Sequences in PCR, RT-qPCR and CHIP-qPCR

Gene	Forward (5'-3')	Reverse (5'-3')
MYC	GGCTCCTGGCAAAAGGTCA	CTGCGTAGTTGTGCTGATGT
FOSL1	GTGGATGGTACAGCCTCATTT	CCAGTTTGTCAGTCTCCTGTTC
CCND1	GATGCCAACCTCCTCAACGA	ACTTCTGTTCCTCGCAGACC
CDKN1A	AGTCAGTTCCTTGTGGAGCC	CATTAGCGCATCACAGTCGC
PLK1	TGACTCAACACGCCTCATCC	GCTCGCTCATGTAATTGCGG
ST8SIA6-AS1	CCATCCATCATGGCCAAAGC	CAGGGTTTCTTCGGTCGTCA
MALAT1	CTTCCTGTGGCAGGAGAGAC	CGCTTGAGATTTGGGCTTTA
GAPDH	GACATCAAGAAGGGGTGAA	TGTCATACCAGGAAATGAGC
PLK1 pro1	GGGAGAAACCCCGAAGGAATT	AGCAGCAGCCTTTAAACCCG
PLK1 pro2	ATTCCTCCAGCAATCAGTTGT	CTGCTTTCCTTCTGGGAGTCA
WT E-box promoter	GGGTACCAGGCTATCCCACGTGTTCG	CAAGCTTCAGCAGCAGCCTTTAAACCC
Mut E-box promoter	GGGTACCAGGCTATCCAACGTGTTCG	CAAGCTTCAGCAGCAGCCTTTAAACCC
