# Supplementary material for: Targeting ST8SIA6-AS1 counteracts KRASG12C inhibitor resistance through abolishing the reciprocal activation of PLK1/c-Myc signaling
Source: Exp Hematol Oncol. 2023 Dec 16;12:105. doi: 10.1186/s40164-023-00466-3 (PMC10724920; doi:10.1186/s40164-023-00466-3)
Supplement: Supplementary file 2 — Additional file 2: Figure S1. C-Myc alteration predicts for the response to KRASG12Ci. Figure S2. C-Myc is vital for KRASG12C-mutant cell proliferation and mediates resistance to KRASG12Ci. Figure S3. The reciprocal activation of PLK1/c-Myc pathway confers resistance to KRASG12Ci. Figure S4. PLK1i synergizes with KRASG12Ci via ERK-dependent and -independent downregulation of c-Myc. Figure S5. ST8SIA6-AS1 promotes malignant proliferation of KRASG12C-mutant cancers through Aurora A/PLK1/c-Myc activation. Figure S6. Targeting ST8SIA6-AS1 reverses cell resistance to KRASG12Ci. [file 40164_2023_466_MOESM2_ESM.pdf]

Targeting ST8SIA6-AS1 counteracts KRAS^{G12C} inhibitor resistance through abolishing the reciprocal activation of PLK1/c-Myc signaling

Yafang Wang[#1], Mingyue Yao[#1 2 3], Cheng Li[1 4], Kexin Yang[4 5], Xiaolong Qin[1 4], Lansong Xu[1 2 3], Shangxuan Shi[1 4], Chengcheng Yu[3 5], Xiangjun Meng[6 7 8], Chengying Xie[1 4 5]

Additional file 2: Supplementary Figures

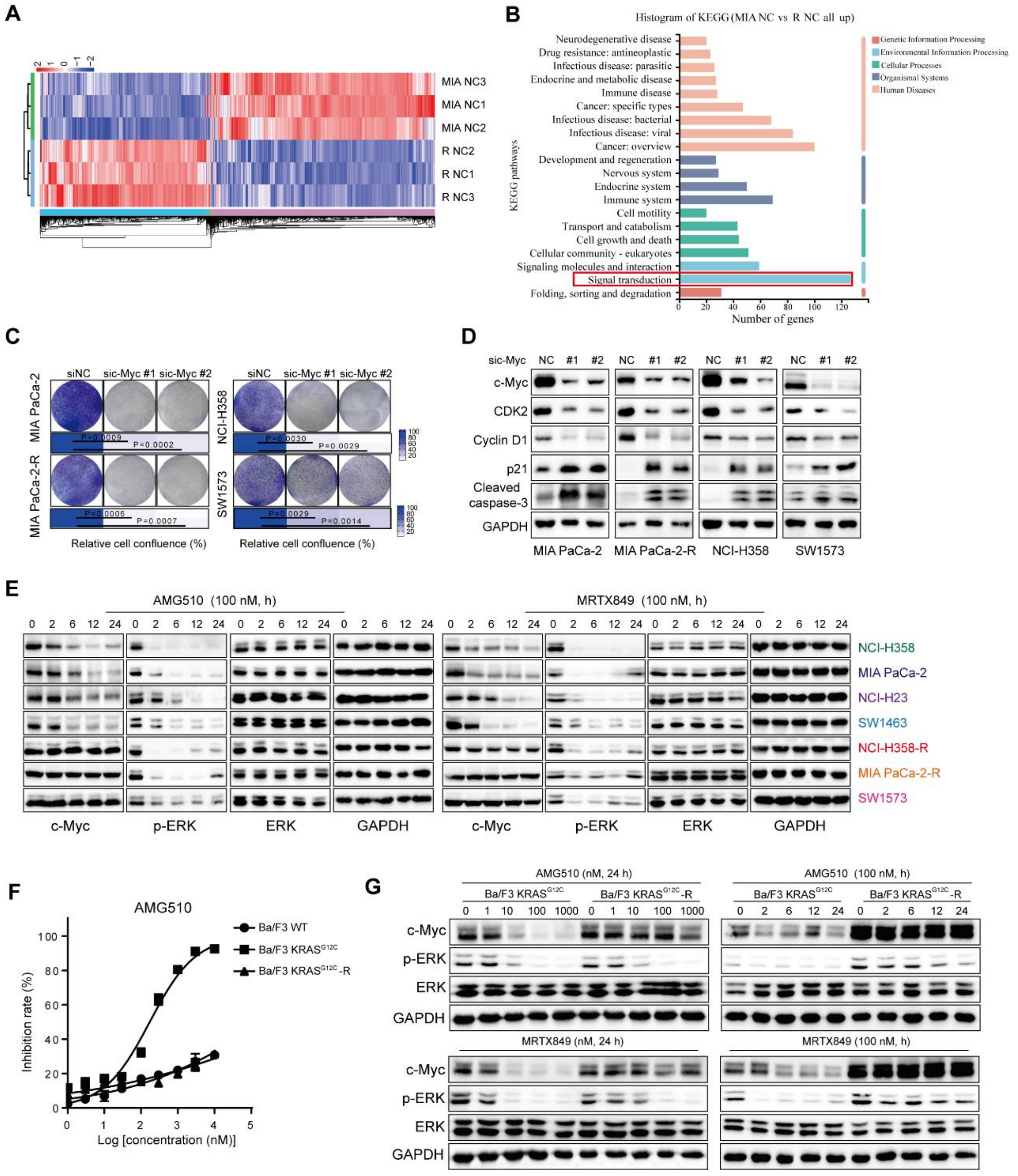

Fig. S1 C-Myc alteration predicts for the response to KRAS^{G12C}i.

A The hierarchical clustering and heatmap showing 3,417 DEGs between MIA PaCa-2 and MIA PaCa-2-R cells. The DEGs were defined as an absolute log2 fold change exceeding 1. **B** KEGG analysis of RNA-seq data from MIA PaCa-2 and MIA PaCa-2-R cells. **C** Cells were transfected with siRNAs against c-Myc or siRNA control (siNC) and cell colony formation ability were tested. **D** Western blot analysis showed the expression of c-Myc targets in c-Myc-knockdown cells. **E** Western blot analysis of $KRAS^{G12C}$-mutant cell lines under the time-dependent treatment of AMG510 or MRTX849. **F** Inhibition rate of cell proliferation in Ba/F3 wild type (WT), Ba/F3 expressing $KRAS^{G12C}$ parental and $KRAS^{G12C}$i-resistant cells treated with increasing concentrations of AMG510. **G** Western blot analysis of Ba/F3 $KRAS^{G12C}$ parental and resistant cell lines under the dose- or time- dependent treatments of AMG510 or MRTX849.

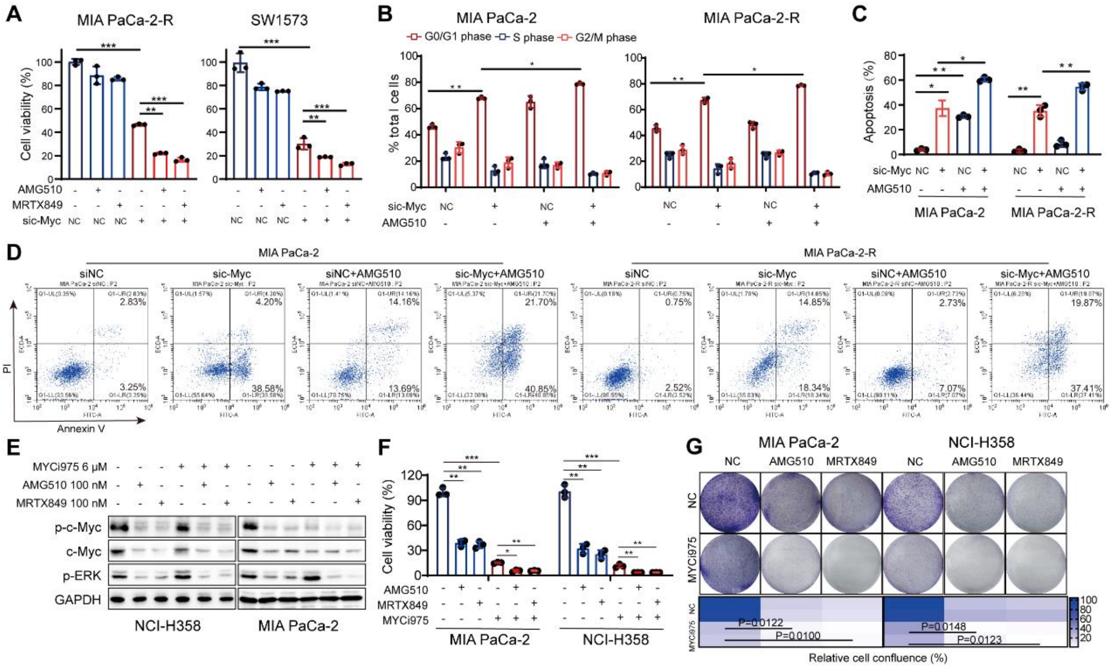

Fig. S2 C-Myc is vital for KRAS^{G12C}-mutant cell proliferation and mediates resistance to KRAS^{G12C}i

A Cells transfected with siNC or sic-Myc were exposed to AMG510 or MRTX849 for 72 h and cell viability were analyzed. Cell cycle (**B**) and cell apoptosis analysis (**C**) of MIA PaCa-2 parental and resistant cells were transfected with siNC or sic-Myc for 48 h and then treated with or without 100 nM AMG510 for 24 h. **D** Flow cytometry data of cell apoptosis in c-Myc-knockdown MIA PaCa-2 parental and resistant cells followed by AMG510 (100 nM, 24 h) treatment. **E** Western blot analysis with the indicated antibodies of protein lysates from NCI-H358 and MIA PaCa-2 cells cultured with KRAS^{G12C}i in combination with MYCi975 for 24 h. **F** Relative cell viability of MIA PaCa-2 and NCI-H358 cells cultured with 10 nM KRAS^{G12C}i in combination with 6 μM MYCi975 for 5 days was analyzed by SRB assay (n = 3). **G** Colony formation of cells cultured with 1 μM KRAS^{G12C}i in combination with 2 μM MYCi975. Data represent the average and SD. Statistical significance was assessed using two-tailed unpaired Student's t test. *P<0.05, **P<0.01, ***P<0.001.

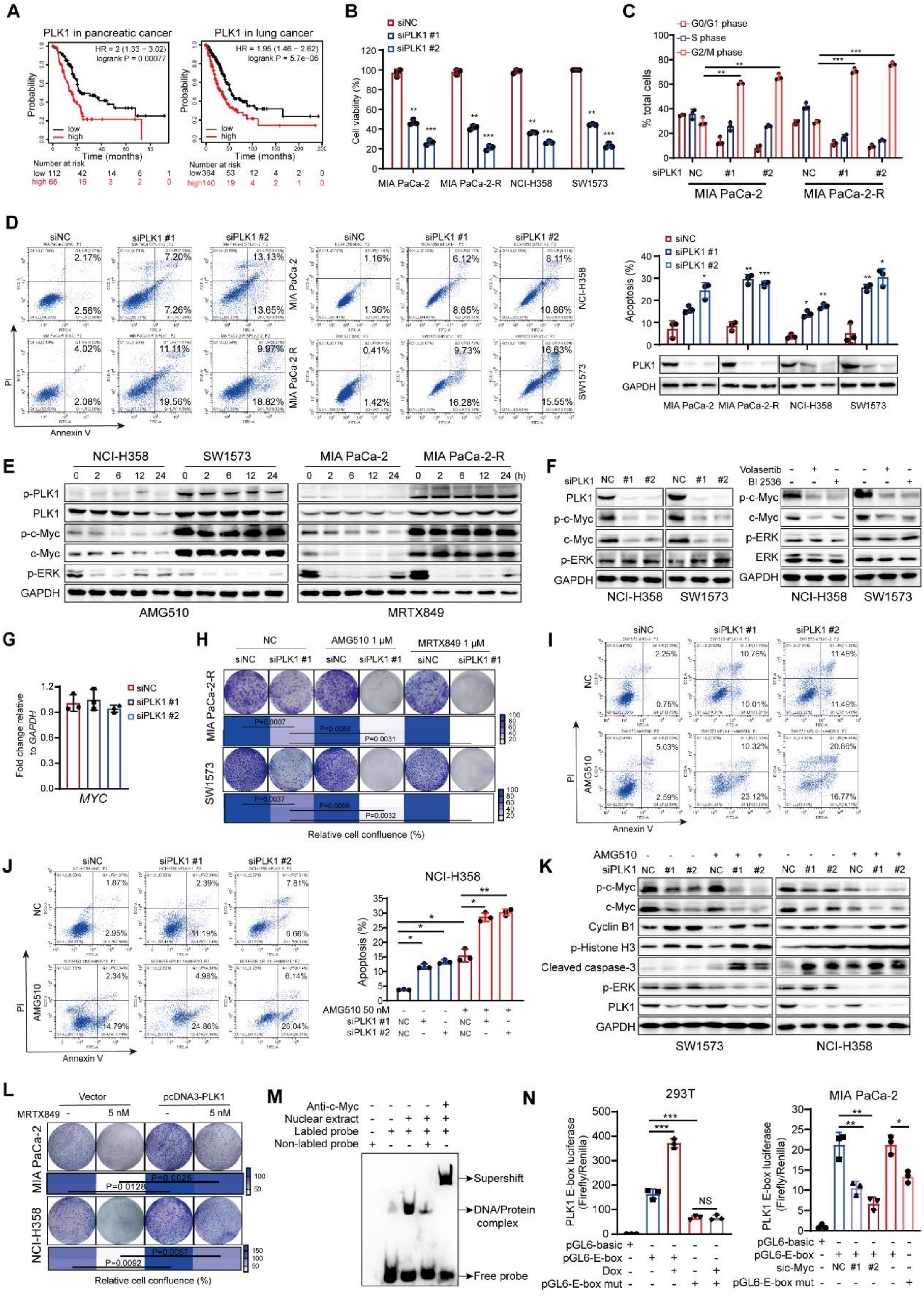

Fig. S3 The reciprocal activation of PLK1/c-Myc pathway confers resistance to KRAS^{G12C}i.

A Kaplan-Meier overall survival for pancreatic cancer and lung cancer patients (TCGA database)

with high or low PLK1 expression. **B** Cells were transfected with siNC or siPLK1 and cell viability were tested after 72 h. Cell cycle (48 h) (**C**) and cell apoptosis (72 h) (**D**) of PLK1-knockdown cells were analyzed by flow cytometry. **E** Western blot analysis with the indicated antibodies of protein lysates from $KRAS^{G12C}$-mutant cells cultured with 100 nM AMG510 or MRTX849 for indicated times. **F** Western blot analysis of cells treated with siPLK1 or 20 nM PLK1i for 48 h. **G** MYC mRNA levels in PLK1-knockdown MIA PaCa-2-R cells. **H** Cell colony formation of siNC- or siPLK1- transfected cells treated with the indicated concentrations of AMG510 or MRTX849. **I** SW1573 cells were transfected with siNC or siPLK1 for 48 h and then treated with 100 nM AMG510 for 24 h. The percentage of annexin V-positive cells were determined by flow cytometry. NCI-H358 cells were transfected with siNC or siPLK1 for 48 h and then treated with 50 nM AMG510 for 24 h. Apoptotic cells were determined by flow cytometry (**J**) and immunoblot analysis was performed (**K**). **L** Cell colony formation of PLK1- or vector- expressing MIA PaCa-2 and NCI-H358 cells treated with MRTX849 (5 nM). **M** Representative super-shift EMSA of c-Myc-responsive E-box element in human *PLK1* promoter using the nuclear extracts of MIA PaCa-2-R cells with or without anti-c-Myc antibody. **N** pGL6 E-box wild-type (WT) or mutant (mut) luciferase constructs were transfected into FUW-tetO-hMYC-293T or MIA PaCa-2 cells and reporter gene expressions were quantified (Firefly luciferase activity normalized to Renilla luciferase activity as an indicator of transfection efficiency). Data represent mean ± SD. Statistical significance was assessed using two-tailed unpaired Student's t test. *P<0.05, **P<0.01, ***P<0.001.

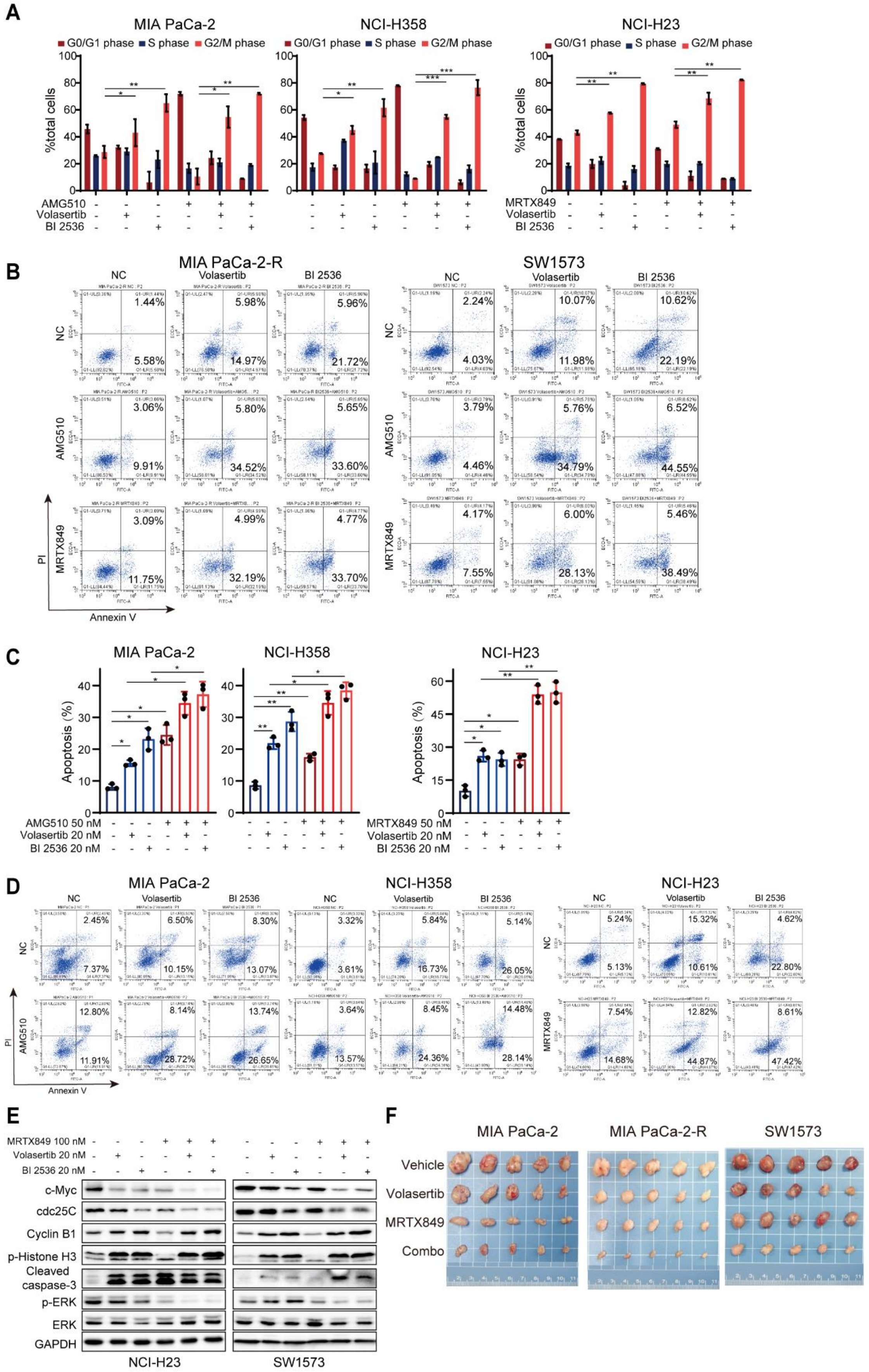

Fig. S4 PLK1i synergizes with KRAS^{G12C}i via ERK-dependent and -independent

downregulation of c-Myc.

A Cell cycle of $KRAS^{G12C}$-mutant cells treated with Volasertib (20 nM) or BI 2536 (20 nM) and $KRAS^{G12C}$i (50 nM) for 24 h. **B** Representative flow cytometry results of apoptosis assay in MIA PaCa-2-R and SW1573 cells treated with PLK1i in combination with $KRAS^{G12C}$i for 24 h. **C, D** Cell apoptosis assay in MIA PaCa-2, NCI-H358 and NCI-H23 cells treated with PLK1i in combination with $KRAS^{G12C}$i for 24 h and representative flow cytometry data were shown. Data are presented as mean ± SD (n = 3). **E** Immunoblotting of NCI-H23 and SW1573 cell lines treated as indicated for 24 h. **F** Images of excised tumors in MIA PaCa-2, MIA PaCa-2-R and SW1573 cell xenografts treated with vehicle, MRTX849 and Volasertib alone or in combination. Statistical significance was assessed using two-tailed unpaired Student's t test. *P<0.05, **P<0.01, ***P<0.001.

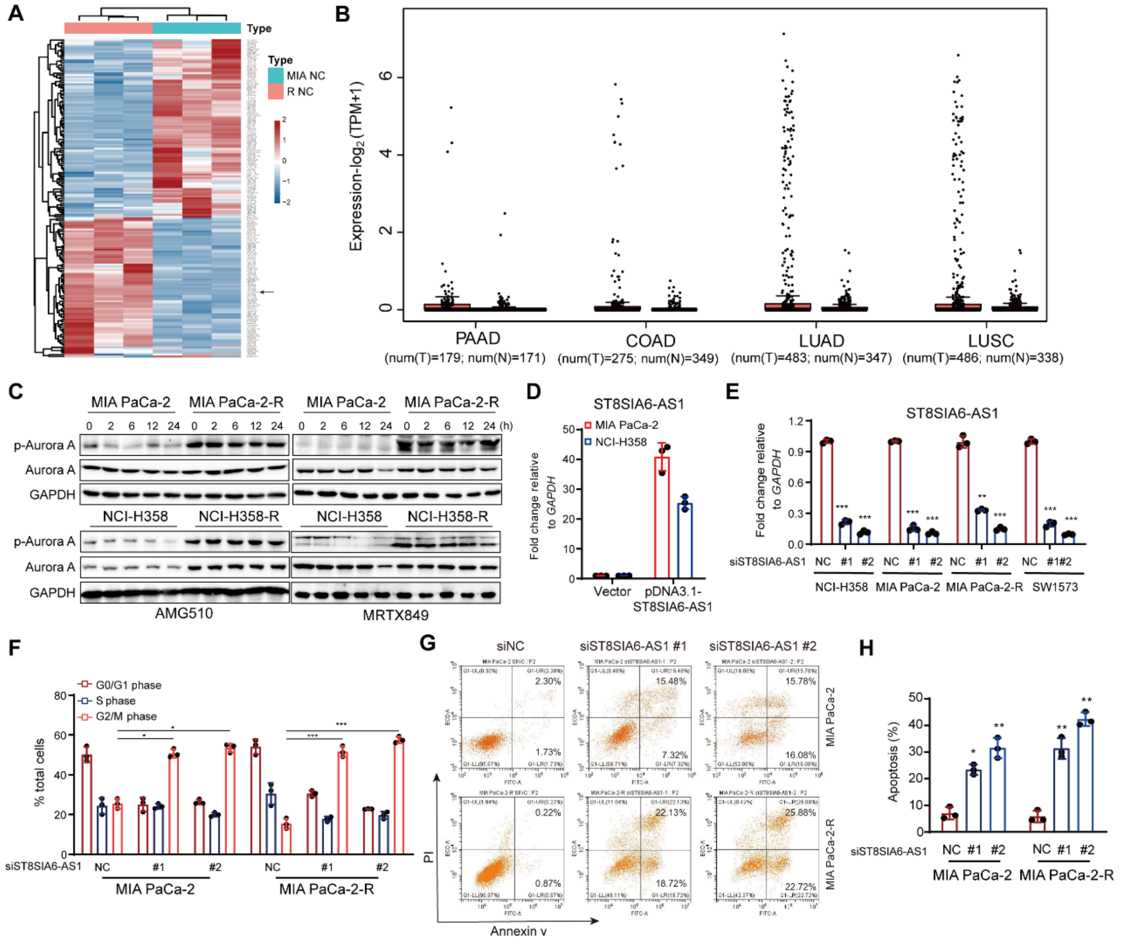

Fig. S5 ST8SIA6-AS1 promotes malignant proliferation of KRAS^{G12C}-mutant cancers through Aurora A/PLK1/c-Myc activation.

A Heatmap showing the differentially expressed lncRNAs in MIA PaCa-2 parental and resistant cells. ST8SIA6-AS1 was pointed by an arrow. **B** ST8SIA6-AS1 expression in the tumors and paired normal tissues of PAAD, colorectal adenocarcinoma (COAD), lung adenocarcinoma (LUAD) and lung squamous cell carcinoma (LUSC) cancer patients from TCGA database. **C** Immunoblot analysis of Aurora A activation in KRAS^{G12C}-mutant cells treated with 100 nM AMG510 or MRXT849 for indicated times. **D** RT-qPCR of ST8SIA6-AS1 levels were determined in cells transfected with plasmid pcDNA3.1-ST8SIA-AS1. **E** RT-qPCR of ST8SIA6-AS1 levels in siRNA-mediated ST8SIA-AS1-depleted cells. **F** ST8SIA-AS1 knockdown cells (48 h) were subjected to flow cytometry for cell cycle analysis. ST8SIA-AS1 knockdown cells (72 h) were subjected to cell

apoptosis analysis. Representative flow cytometry data were shown (**G**) and analyzed (**H**). Data represent mean ± SD (n=3). Statistical significance was assessed using two-tailed unpaired Student's t test. *P<0.05, **P<0.01, ***P<0.001.

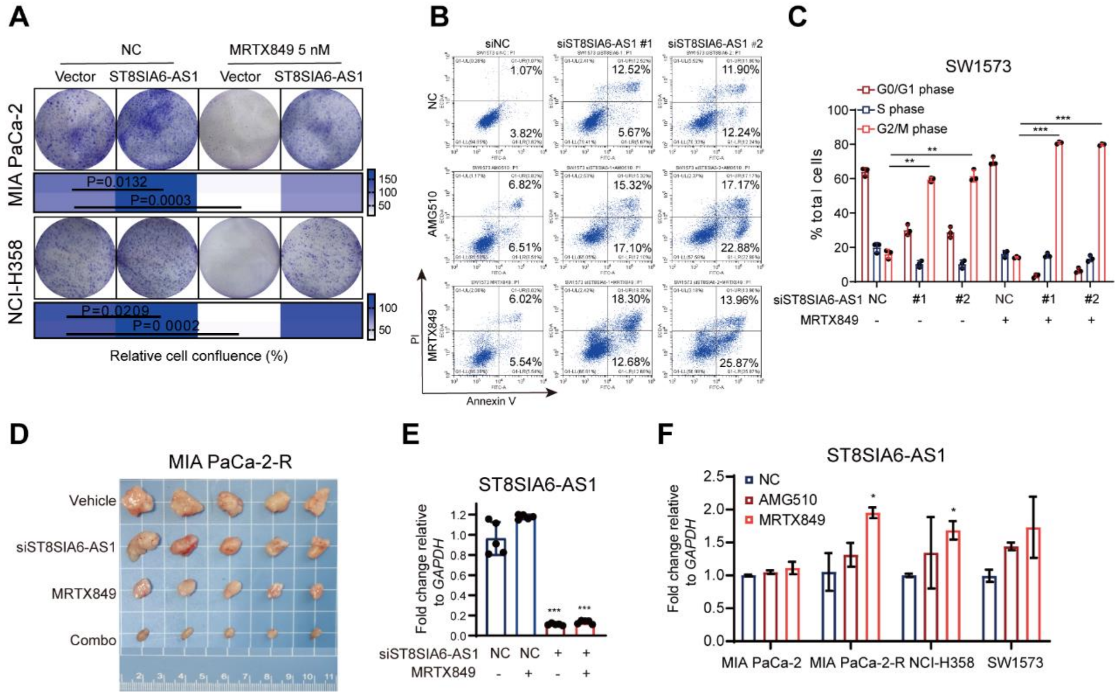

Fig. S6 Targeting ST8SIA6-AS1 reverses cell resistance to KRAS^{G12C}i.

A Cell colony formation assay of ST8SIA6-AS1-overexpressed MIA PaCa-2 and NCI-H358 cells treated with the indicated concentration of MRTX849. **B** Representative flow cytometry data of cell apoptosis analysis in ST8SIA6-AS1-knockdown SW1573 cells treated with or without 100 nM KRAS^{G12C}i for 24 h. **C** Cell cycle in ST8SIA6-AS1-knockdown SW1573 cells treated by 100 nM MRTX849 for another 24 h. **D** Images of excised tumors in MIA PaCa-2-R xenografts treated with vehicle, MRT849 and siST8SIA6-AS1 alone or in combination. **E** RT-qPCR analysis of ST8SIA6-AS1 levels in MIA PaCa-2-R xenografts treated with vehicle, MRTX849 and ST8SIA6-AS1 siRNA, alone or in combination. **F** The levels of ST8SIA6-AS1 in cells treated with 100 nM KRAS^{G12C}i for 24 h were tested by RT-qPCR. Data are shown as mean ± SD (error bars). *P<0.05, **P<0.01, ***P<0.001.
